# A Transcriptomic Analysis of *Echinococcus granulosus* Larval Stages: Implications for Parasite Biology and Host Adaptation

**DOI:** 10.1371/journal.pntd.0001897

**Published:** 2012-11-29

**Authors:** John Parkinson, James D. Wasmuth, Gustavo Salinas, Cristiano V. Bizarro, Chris Sanford, Matthew Berriman, Henrique B. Ferreira, Arnaldo Zaha, Mark L. Blaxter, Rick M. Maizels, Cecilia Fernández

**Affiliations:** 1 Program in Molecular Structure and Function, Hospital for Sick Children, University of Toronto, Toronto, Canada; 2 Cátedra de Inmunología, Facultad de Química, Universidad de la República, Montevideo, Uruguay; 3 Laboratório de Biologia Molecular de Cestódeos and Laboratorio de Genômica Estrutural e Funcional, Centro de Biotecnologia, Universidade Federal do Rio Grande do Sul, Porto Alegre, Brazil; 4 Parasite Genomics, The Wellcome Trust Sanger Institute, Hinxton, United Kingdom; 5 Institute of Evolutionary Biology, School of Biological Sciences, University of Edinburgh, Edinburgh, United Kingdom; 6 Institute of Immunology and Infection Research, School of Biological Sciences, University of Edinburgh, Edinburgh, United Kingdom; University of Queensland, Australia

## Abstract

**Background:**

The cestode *Echinococcus granulosus* - the agent of cystic echinococcosis, a zoonosis affecting humans and domestic animals worldwide - is an excellent model for the study of host-parasite cross-talk that interfaces with two mammalian hosts. To develop the molecular analysis of these interactions, we carried out an EST survey of *E. granulosus* larval stages. We report the salient features of this study with a focus on genes reflecting physiological adaptations of different parasite stages.

**Methodology/Principal Findings:**

We generated ∼10,000 ESTs from two sets of full-length enriched libraries (derived from oligo-capped and *trans*-spliced cDNAs) prepared with three parasite materials: hydatid cyst wall, larval worms (protoscoleces), and pepsin/H^+^-activated protoscoleces. The ESTs were clustered into 2700 distinct gene products. In the context of the biology of *E. granulosus*, our analyses reveal: (i) a diverse group of abundant long non-protein coding transcripts showing homology to a middle repetitive element (EgBRep) that could either be active molecular species or represent precursors of small RNAs (like piRNAs); (ii) an up-regulation of fermentative pathways in the tissue of the cyst wall; (iii) highly expressed thiol- and selenol-dependent antioxidant enzyme targets of thioredoxin glutathione reductase, the functional hub of redox metabolism in parasitic flatworms; (iv) candidate apomucins for the external layer of the tissue-dwelling hydatid cyst, a mucin-rich structure that is critical for survival in the intermediate host; (v) a set of tetraspanins, a protein family that appears to have expanded in the cestode lineage; and (vi) a set of platyhelminth-specific gene products that may offer targets for novel pan-platyhelminth drug development.

**Conclusions/Significance:**

This survey has greatly increased the quality and the quantity of the molecular information on *E. granulosus* and constitutes a valuable resource for gene prediction on the parasite genome and for further genomic and proteomic analyses focused on cestodes and platyhelminths.

## Author Summary

Cestodes are a neglected group of platyhelminth parasites, despite causing chronic infections to humans and domestic animals worldwide. We used *Echinococcus granulosus* as a model to study the molecular basis of the host-parasite cross-talk during cestode infections. For this purpose, we carried out a survey of the genes expressed by parasite larval stages interfacing with definitive and intermediate hosts. Sequencing from several high quality cDNA libraries provided numerous insights into the expression of genes involved in important aspects of *E. granulosus* biology, e.g. its metabolism (energy production and antioxidant defences) and the synthesis of key parasite structures (notably, the one exposed to humans and livestock intermediate hosts). Our results also uncovered the existence of an intriguing set of abundant repeat-associated non-protein coding transcripts that may participate in the regulation of gene expression in all surveyed stages. The dataset now generated constitutes a valuable resource for gene prediction on the parasite genome and for further genomic and proteomic studies focused on cestodes and platyhelminths. In particular, the detailed characterization of a range of newly discovered genes will contribute to a better understanding of the biology of cestode infections and, therefore, to the development of products allowing their efficient control.

## Introduction

Cestodes are a major group of helminths infecting humans and domesticated animals, of global sanitary and economic importance [1] and include the parasites responsible for echinococcosis [2] and cysticercosis [3]. While genomic initiatives are now well advanced for some of these organisms [4], and proteomic analyses have recently been carried out [5], [6], [7], our knowledge at the transcriptomic level remains limited. We selected *Echinococcus granulosus* as a suitable target for analysis of gene expression by key life cycle stages.

*E. granulosus* is the agent of cystic echinococcosis, a major zoonosis that affects humans and a wide range of domestic and wild animals worldwide [8], [9]. Control efforts have had little global impact and the infection remains highly endemic in the Southern Cone of Latin America (Argentina, Chile, Uruguay, Southern Brazil and Peru), as well as in large areas of Asia and Africa, and in patches of Europe and North America [10]. Although difficult to assess due to underreporting, the disease has a substantial global burden, which is estimated at over 1 million DALYs per year [11].

The *E. granulosus* life cycle involves two mammalian hosts. The intermediate hosts (ungulates and, accidentally, humans) ingest eggs that develop into a hydatid cyst containing larval worms or protoscoleces (PS), bathed in hydatid fluid that includes parasite as well as host proteins. The PS are clearly differentiated into distinct tissues (the rostellar pad, the neck, the suckers and the body; [12]), and the hydatid cyst is delimited by a cyst wall (CW), consisting of an inner germinal layer of metabolically active parasite cells and an outer protective acellular mucin-rich laminated layer [13], which appears to be evolutionarily optimized for eliciting non-inflammatory responses from the host immune system [14]. The cyst is usually surrounded by a host-derived collagen capsule, the adventitial layer. Infection in the definitive host (always a canid) arises from ingestion of PS encysted in the viscera of the intermediate hosts. PS are activated by contact with stomach acid and enzymes, which can be reproduced in the laboratory by exposure to pepsin at low pH. In the duodenum, they develop into adult tapeworms that can reside for long periods, indicating that PS establishment requires modulation of the host immune response [15], [16]. In addition, *E. granulosus* has a fascinating alternate reverse development, as PS escaping from a ruptured cyst in an intermediate host are able to differentiate asexually into secondary hydatid cysts (reviewed by [17]).

To study the molecular basis of the host-parasite interaction, and to gain understanding of *E. granulosus* developmental and metabolic aspects, we have analyzed the transcriptomes from the CW, the resting PS (*i.e.* as present in the hydatid cyst) and pepsin/H^+^-activated PS (PSP). We previously reported a new method to construct full-length cDNA libraries by an oligo-capping method [18]. Because some *E. granulosus* mRNAs bear a *trans*-spliced leader (SL) sequence [19], which blocks oligo-capping [18], in this study we have analyzed both oligo-capped and SL-bearing transcripts to ensure that we also captured genes that are processed by *trans*-splicing. Transcriptome analyses focusing on other parasitic platyhelminth species have been published, including the trematodes *Schistosoma mansoni* [20], *Schistosoma japonicum* [21], *Clonorchis sinensis* [22], *Opisthorchis viverrini* [23] and *Fasciola hepatica* [24], and the cestodes *Mesocestoides corti* (syn. *vogae*) [25], *Echinococcus multilocularis* [26], *Moniezia expansa* [27], and *Taenia solium* [28], [29], [30]. The recent availability of high throughput sequencing technologies has also stimulated transcriptome surveys of various adult liver flukes [31], and the cestode *Taenia pisiformis* [34].

We report the analysis of 9,452 ESTs from ∼2,700 distinct genes, generated from *E. granulosus* larval stages. These data represent about 20% of the estimated 11,000 protein-coding genes of the parasite [4]. In addition, they reveal the expression of remarkably abundant putatively non-protein-coding transcripts (ncRNAs) that could either be active by themselves as long ncRNAs or represent precursors of small RNAs. The full genome sequence of *E. granulosus*, now nearing completion [4], together with the transcriptomic data presented here will constitute invaluable resources to deepen our understanding of the biology of this parasite.

## Materials and Methods

### Source of parasite material and preparation of cDNA libraries

*E. granulosus* PS and CW (germinal and laminated layers) were recovered under aseptic conditions from hydatid cysts of the G1 genotype, present in the lungs of naturally infected bovines in Uruguay. Cysts were collected during the routine work of local abattoirs in Montevideo (Uruguay). The G1 genotype, the common ‘sheep strain’ which infects cattle in areas of intense sheep farming, has recently been reclassified into *E. granulosus sensu stricto* (that also includes G2 and G3; [35], [36]); it has a worldwide distribution and its presence coincides with high prevalence of human infection [9]. PS and CW were stored at −80°C in Trizol reagent (GibcoBRL) until RNA extraction. One fraction of freshly isolated PS was incubated with pepsin prior to treatment with Trizol (PSP). The processing of parasite materials and the construction of cDNA libraries were previously described in detail [18]. In brief, two sets of full-length enriched libraries were prepared using total RNA from the three materials (CW, PS and PSP). RNA from each source was reverse transcribed with a tagged oligo-dT. In the first set of libraries, full-length mRNAs were ligated to a 5′oligo, permitting PCR amplification of the intact mRNA population (oligo-capped (GR) libraries). In the second set, a 5′primer for the *E. granulosus* SL sequence [19]) was used (SL libraries).

### Library sequencing

The libraries were plated out and random colonies picked for EST sequencing. A small-scale analysis (5′first-pass sequencing) was initially carried out on AB3730 instruments (Applied Biosystems) in the GenePool Facility (Edinburgh), on about 250 randomly isolated clones from each library, as previously described [18]. Further sequencing from these libraries was performed at the Sanger Institute and the Centro de Biotecnologia in MegaBace 1000 instruments (Amersham Biosciences). An alkaline lysis method for plasmid DNA preparation in 96-well plates was used; plasmid DNA was subsequently purified through Millipore plates and resuspended in 30 µl of MilliQ water. 5′ and 3′ ESTs were carried out from each plasmid, using 500 ng of DNA and the DYEnamic ET Terminator Kit (Amersham Biosciences), according to the instructions of the manufacturer.

### Bioinformatics

Sequence processing was performed using the PartiGene pipeline [37]. Raw sequence trace data was processed to remove low quality, vector, host (bovine), linking and poly(dA) sequences. For annotation purposes, each sequence was subject to a BLASTN search against the non-redundant DNA database [38] as well as a BLASTX search against the non-redundant protein database [39]. Sequences have been submitted to dbEST [40]. Sequences were collated and clustered on the basis of BLAST similarity to derive groups of sequences, which putatively derive from the same gene using the software package - CLOBB [41]. These groups were then used to derive a set of consensus sequences using the freely available software package PHRAP (P. Green unpublished data). It is worth noting that, while the CLOBB clustering tool attempts to minimize the generation of chimeric consensi, transcripts representing alternative splice forms may be clustered into separate groups whereas members of the same gene family can be merged into the same group [41]. This set of consensus sequences together with those groups containing only a single sequence (‘singletons’) form a non-redundant set of gene sequences, which we refer to as a partial genome. The corresponding *E. granulosus* dataset is available from PartiGeneDB (http://www.compsysbio.org/partigene/annotation/viewset.php). For comparative purposes, we also performed TBLASTX comparisons against: 1) a set of 688 eukaryotic partial genomes in our in-house partial genome database (PartiGeneDB - [42]); 2) a set of 3,178 non-redundant (clustered) sequences derived from 12,483 ESTs generated from *E. multilocularis* (K. Brehm and C. Fernández, personal communication); and 3) a set of 2,271 non-redundant (clustered) sequences derived from 3,947 ESTs generated from *Fasciola hepatica* (M. Berriman, personal communication).

Peptide predictions were performed using the prot4EST software [43]. Domain and signal peptide predictions were obtained using PFAM [44] and SignalP V3.0 [45], respectively. Similarity analyses comparing peptides among three different datasets were performed using the SimiTri comparison tool [46]. Alignments were initially created using ClustalW2 [47] and refined manually. Analyses of the presence of putative *O*-glycosylation sites, signals for GPI incorporation and transmembrane helices were carried out with the tools available at the ExPASy Proteomics Server (http://expasy.org/proteomics): NetOGlyc, PI predictor and TMHMM, respectively. Putative platyhelminth orthologs of *E. granulosus* cDNAs were identified using BLAST by applying the best-reciprocal-hits approach [48]. For the phylogenetic analysis of identified tetraspanins, an alignment was manually refined taking into account the consensus of 6-Cys-a and 8-Cys-a cysteine patterns (adapted from [49] and [50]) and used to construct a minimum evolution phylogenetic tree using MEGA 4 [51] with default parameters. Bootstrap values were expressed as percentage of 1000 replicates and were considered significant if >50%.

## Results and Discussion

### Stage specific gene expression is a clear feature of the *E. granulosus* transcriptome

A total of 9,462 ESTs (7722 5′ESTs and 1740 3′ESTs) were generated from six full-length enriched *E. granulosus* cDNA libraries constructed from three sources of parasite material: CW, PS and PSP. These represent key stages in the parasite life cycle that interface with either the intermediate host (mainly the CW, during the chronic phase of infection) or the definitive host (mainly PSP, at the onset of infection). The boundaries between stages are not absolute, and each preparation should be considered as ‘highly enriched’ in transcripts from the corresponding stage. For example, the CW from a healthy cyst usually contains some PS, and pepsin/H^+^ treatment does not activate all PS in a sample because their development inside the cyst is not synchronous.

Following strategies targeted at cloning cDNAs with an intact 5′ end, we constructed two sets of libraries, either by exploiting the 5′ *trans*-spliced leader sequence (SL libraries) [52] or by using an oligo-capping method based on the GeneRacer protocol (GR libraries) to select full length cDNAs [53]. The two library construction methods produced sequences of similar length (**Table 1**). After processing, the dataset gave 2,700 putative genes comprised of 1,328 clusters containing more than one sequence and 1,372 ‘singletons’ (see *E. granulosus* dataset at PartiGeneDB: http://www.compsysbio.org/partigene/annotation/viewset.php) (**Table 1**). A total of 166 putative genes (23 clusters and 143 singletons) were derived from 3′ESTs only. Taking into account the library construction strategies and that a majority of ESTs were carried out from the 5′end, this number provides an (over)estimated maximum of the transcripts that could correspond to non-overlapping regions of the same gene.

**Table 1:** Sequence summary table.

Library	Number of sequences	Number of singletons	Number of clusters	Redundancy	Library specific clusters	Average length of sequences (bp)
CWSL	1851	238	469	2.6	103	481+/−93
CWGR	1220	215	314	2.3	106	579+/−140
PSSL	1383	145	367	2.7	65	448+/−167
PSGR	1482	199	392	2.5	104	529+/−136
PSPSL	1886	385	504	2.1	77	466+/−92
PSPGR	1640	190	387	2.8	104	478+/−136
ALL:GR	4342	604	708	3.3	568	524+/−143
ALL:SL	5120	768	760	3.4	620	467+/−118
ALL	9462	1372	1328	3.5		493+/−133

Clusters including 3′ESTs (1740 sequences): 143 singletons; and 717 clusters (of which 694 also contain 5′ESTs, and 23 3′ESTs only).

Clusters including ESTs from libraries of only one stage (‘stage-specific clusters’): CW-specific clusters, 226; PS-specific clusters, 173; PSP-specific clusters, 189.

**Figure 1:**
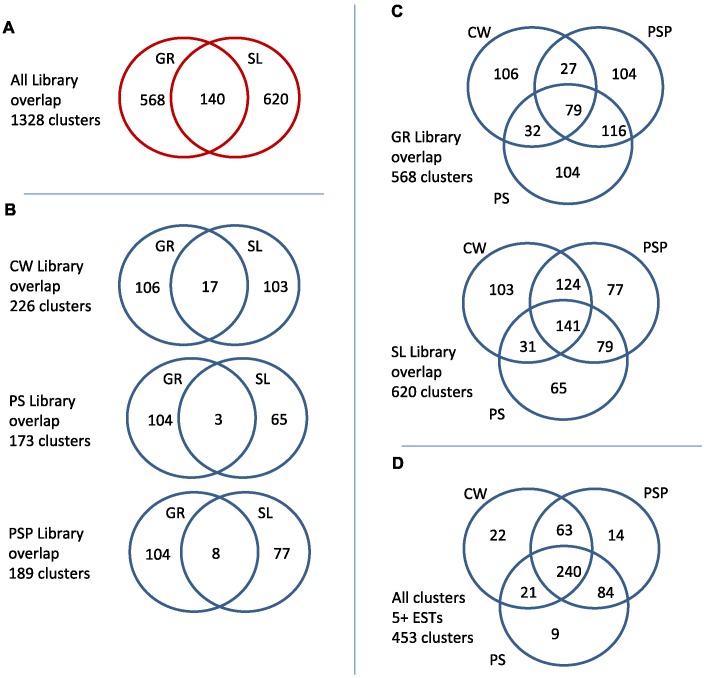
Library overlap. The Venn diagrams show the overlap in cluster membership between various libraries sequenced in this study. (A) Only 140 of the 1,328 generated clusters contained members from both GR- and SL-derived libraries. (B) and (C) show the overlap between libraries prepared from various parasite materials. (D) When we consider all clusters containing five or more ESTs, we note a larger overlap across the three stages, suggesting that a lack of overlap elsewhere may be due to sampling biases.

The distribution of the clusters according to the parasite stage and also the type of cDNA library in which they were found are summarized in **Figure 1**. The GR and SL libraries were largely non-overlapping as expected from previous work [18], with only ∼10.5% (140/1328) of clusters comprising reads from both types (**Figure 1A and B**). The lack of overlap between GR and SL libraries is due to the fact that the GR oligo rarely ligates to the 5′ SL, likely because of some structural feature of the *Echinococcus* SL (perhaps the formation of a short hairpin loop, as was recently proposed [54]).

In both GR and SL library datasets, the proportion of clusters associated with only one stage (‘stage-specific clusters’) was considerable (**Figure 1C**). For example, 43% of hydatid cyst wall GR clusters (106/244) were not found in other stages, and 26% of hydatid cyst wall SL clusters (103/399) were similarly stage-specific. In addition, 44% (332/747) of clusters involving PSP in GR and SL libraries, were absent from the untreated PS sample. The high level of stage-specific expression may reflect the sharply contrasting environments and developmental programs associated with the different stages. On the other hand, as we have not sampled the transcriptome to exhaustion, some of these differences are more likely due to limited sampling rather than to differential gene expression. In fact, a much greater overlap between libraries was noted when considering clusters derived from five or more sequences (**Figure 1D**; see also next section).

### Most abundant transcripts highlighted common as well as distinct features of each developmental stage

**Table 2** presents the most highly represented transcripts from each analyzed stage (CW, PS and PSP). Surprisingly, the most highly abundant transcripts in the three parasite stages (EGC00310 and EGC03058) were non-protein coding RNAs (ncRNAs) showing similarity to the *E. granulosus* repetitive DNA element, EgBRep [55]. As described in more detail below, these molecules are closely related and can be regarded as a single cluster with micro-variation. Interestingly, a separate cluster showing similarity to EgBRep was largely PS specific and, in contrast to the previous ones, derived from *trans*-spliced cDNAs (EGC02791).

**Table 2:** Most abundant transcripts in each stage.

Stage	Cluster ID – Blast similarity to UniProt/EMBL	No ESTs	Library	CW	PS	PSP
CW	EGC00310 - X67152.1 - *E. granulosus* EgBRep repetitive element (blastn)	259	GR	74	108	74
			SL	1	1	1*
	EGC00548 - C4Q877 - *S. mansoni* [Smp_144420] hypothetical protein	98	GR	–	–	–
			SL#	50	20	28
	EGC03058 - X67152.1 - *E. granulosus* EgBRep repetitive element (blastn)	122	GR	47	45	30
			SL	–	–	–
	EGC00317 - No significant hit	37	GR	37	–	–
			SL	–	–	–
	EGC00373 - Q0PH42 - *T. solium* SLC10	85	GR	–	–	1*
			SL#	32	31	21
	EGC00290 - B6VFH3 - *E. multilocularis* tetraspanin TSP-1	29	GR	29	–	–
			SL	–	–	–
	EGC00843 - Q5DDJ8 - *S. japonicum* SJCHGC05178 [cwf18 splicing factor]	39	GR	27	5	7
			SL	–	–	–
	EGC00369 - Q9GP32 - *E. multilocularis* fructose biphosphate aldolase	44	GR	3	1	–
			SL§	23	7	10
	EGC00857 - Q8MPE9 - *T. solium* putative proteasome maturation protein	51	GR	–	–	–
			SL#	25	11	15
	EGC00435 - D2V1P1 - *Naegleria gruber*i RING finger domain-containing prot.	60	GR	1*	–	–
			SL§	24	15	20
PS	EGC00310 - X67152.1 - *E. granulosus* EgBRep repetitive element (blastn)	259	GR	74	108	74
			SL	1	1	1*
	EGC03058 - X67152.1 - *E. granulosus* EgBRep repetitive element (blastn)	122	GR	47	45	30
			SL	–	–	–
	EGC00366 - Q8MPE3 - *T. solium* putative vacuolar ATPase associated protein	62	GR	–	–	–
			SL#	17	37	8
	EGC01072 - A7SKC2 - *Nematostella vectensis* predicted [ubiquitin-like]	40	GR	–	–	–
			SL§	5	32	3
	EGC02791 - X67152.1 - *E. granulosus* EgBRep repetitive element (blastn)	33	GR	1	–	–
			SL	–	32	–
	EGC00373 - Q0PH42 - *T. solium* SLC10	85	GR	–	–	1*
			SL#	32	31	21
	EGC00647 - Q66KU8 - *Xenopus laevis* MGC85413 protein [cox17]	41	GR	–	1	–
			SL§	10	22	8
	EGC00446 - B6VFH3 - *E. multilocularis* tetraspanin TSP-1	34	GR	–	21	13
			SL	–	–	–
	EGC00548 - C4Q877 - *S. mansoni* [Smp_144420] hypothetical protein	98	GR	–	–	–
			SL#	50	20	28
	EGC00658 - A7SC54 - *Nematostella vectensis* predicted [Cupin_2]	44	GR	–	–	–
			SL#	13	19	12
	EGC00524 - B3DFV0 - *Dario rerio* UPF0631 protein C17orf108 homolog	28	GR	–	–	1
			SL§	3	19	5
PSP	EGC00310 - X67152.1 - *E. granulosus* EgBRep repetitive element (blastn)	259	GR	74	108	74
			SL	1	1	1*
	EGC03058 - X67152.1 - *E. granulosus* EgBRep repetitive element (blastn)	122	GR	47	45	30
			SL	–	–	–
	EGC00548 - C4Q877 - *S. mansoni* [Smp_144420] hypothetical protein	98	GR	–	–	–
			SL#	50	20	28
	EGC00474 - Q86E46 - *S. japonicum* SJCHGC06675 ribosomal protein L16	39	GR	1	11	27
			SL	–	–	–
	EGC00350 - C4Q1G6 - *S. mansoni* 40S ribosomal protein S15	29	GR	3	2	24
			SL	–	–	–
	EGC00553 - C4PYS1 - *S. mansoni* inositol polyphosphate multikinase	56	GR	–	–	–
			SL#	23	10	23
	EGC00370 - C4QLX9 - *S. mansoni* protein [Smp_092500] thioredoxin-like	52	GR	–	–	–
			SL§	18	12	22
	EGC00373 -Q0PH42 - *T. solium* SLC10	85	GR	–	–	1*
			SL#	32	31	21
	EGC00467 - Q15ER7 - *S. mansoni* 60S ribosomal protein L14	34	GR	2	11	21
			SL	–	–	–
	EGC00435 - D2V1P1 - *Naegleria gruberi* RING finger domain-containing prot.	60	GR	1*	–	–
			SL	24	15	20
	EGC00522 - Q8MPE4 - *T. solium* putative NADH ubiquinone oxidoreductase	56	GR	–	–	–
			SL#	18	18	20
	EGC00396 - B0XE28 - *Culex quinquefasciatus* putative protein – COX6B	44	GR	–	–	–
			SL#	12	12	20

* cDNA includes the SL at the 5′end.

SL AUG is:

# in frame with predicted ORF;

§ not in frame with predicted ORF (Table S1).

All other highly expressed transcripts coded for proteins, most of which showed similarity to sequences from other platyhelminths. The CW expressed two stage-specific transcripts at high levels: a novel sequence coding for a putative apomucin (EGC00317) and a member of the tetraspanin family (EGC00290). Interestingly, a further tetraspanin-containing transcript (EGC00446) was restricted to the PS and PSP stages (see below). The remaining highly expressed clusters corresponded to transcripts represented in the three stages but showing some stage bias in the number of ESTs. It is noteworthy that the majority (12/16) corresponded to *trans*-spliced cDNAs, including enzymes participating in energy metabolism (notably, EGC00369, fructose biphosphate aldolase, highly abundant in the CW) and antioxidant systems (EGC00370, thioredoxin-like, abundant in the three stages). The cDNAs that were not *trans*-spliced comprised three ribosomal proteins, prominent in PSP (EGC00474, EGC00350 and EGC00467); and a putative splicing factor, highly expressed in the CW (EGC00843).

Four SL-bearing transcripts encoding hypothetical proteins were amongst the most highly expressed; two of them in all three stages (EGC00548 and EGC00373) and two in PS (EGC00658; EGC00524). Given that high levels of expression are often indicative of essential roles, these represent interesting targets for further investigation.

Consideration of all clusters (see **Table S1**) reinforced these observations; in fact, clusters representing highly expressed transcripts (≥20 ESTs) included: non-protein coding RNAs (EGC02905; EGC00351; EGC00637 and EGC01002), abundant in GR libraries; and mRNAs coding for lactate dehydrogenase (EGC00284), another enzyme from the glycolytic pathway, that predominated in CW; and several ribosomal proteins (EGC00595; EGC00605; EGC00634; EGC01107) in PSP. In addition, a protein containing a dynein light chain domain (EGC00319), immunolocalized to the PS tegument and the germinal layer (EgTeg; [56]) and detected in cyst fluid, PS and germinal layer [6], was highly expressed in all stages, mainly in PSP and CW (see also next section).

### Domain analyses revealed lineage-specific domain expansions

**Figure 2:**
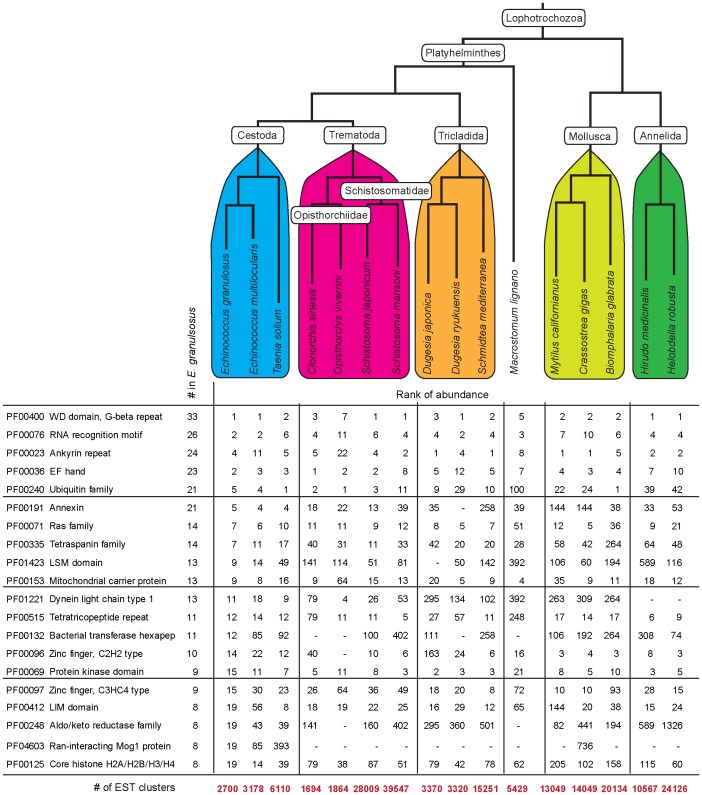
Ranked abundance of PFAM domains across platyhelminth datasets. For each sequence dataset, we determined the incidence of PFAM domains and show the top 20 most abundant domains in our *E. granulosus* dataset. In addition, we provide the relative rank of abundance for an additional ten platyhelminths, as well as five other lophotrochozoans. Sixteen clusters were identified as containing the Tetraspanin domain (PF00335) in our dataset but two of them corresponded to incompletely processed forms of other clusters; this is why only fourteen were considered for the rank (see also **Table 8**). The platyhelminth EST datasets were derived from cDNA libraries of the following materials: PS and metacestode tissue from *E. multilocularis*; larva and adult from *T. solium*; adult from *C. sinensis*, *O viverrini*, *D. ryukuensis* and *M. lignano*; most stages over the life cycles of *S. japonicum* and *S. mansoni*; head from *D. japonica*; juvenile and sexually mature hermaphrodites, and whole body of unspecified stage from *S. mediterranea*. Details of the libraries are available at PartiGeneDB (http://www.compsysbio.org/partigene) from the dataset of each organism.

From the 2,700 clusters identified, we were able to derive 2,584 peptide predictions which were each scanned for putative PFAM domains [44]. Overall, 1,034 domains, representing 193 unique domains, were identified in 808 peptides, as detailed in **Table S1**. **Figure 2** shows the most abundant domains identified within the dataset. We compared the abundance of each PFAM domain relative to EST datasets obtained from ten additional platyhelminths and five other lophotrochozoans. Even though care must be taken while interpreting the data because all sets are partial, this type of comparisons provides a first glimpse into species differences (see *e.g.* [57], [58]).

In fact, despite the datasets differing in size and the diversity of stages used (see legend to **Figure 2** for details), some interesting trends emerged. Four of the top five domains were consistently abundant across the Lophotrochozoa: WD domain (PF00400); RNA recognition motif (PF00076); ankyrin repeat (PF00023) and EF hand (PF00036), as were also the Ras family (PF00071); mitochondrial carrier protein (PF00153); and tetratricopeptide repeat (PF00515).

Relative to other species, the protein kinase domain (PF00069) was relatively poor within both *Echinococcus* species. Conversely, the tetraspanin domain (PF00335) was expanded in platyhelminths; *E. granulosus* proteins identified as containing this domain are analyzed further below. In addition, both trematode and cestode lineages showed expansion in the dynein light chain domain (PF01221), whereas the annexin (PF00191) and Like-Sm ribonucleoprotein (LSM; PF01423) domains appeared expanded only in the cestode lineage. Two of these domains (dynein light chain and annexin) are associated with cellular organization and the third one (LSM) with RNA metabolism.

Thirteen predicted polypeptides (mostly from PS and PSP libraries) contained the dynein light chain domain, involved in intracellular motility of vesicles and organelles along microtubules [59]. Six predicted proteins contained up to four annexin domains; some being highly represented in the CW (EGC00693) or the PSP (EGC00359) stages. The annexins (or lipocortins) are eukaryotic calcium-dependent phospholipid-binding proteins implicated in multiple functions, including exocytosis and endocytosis, signal transduction, and extracellular matrix organization [60].

Thirteen predicted polypeptides encoded by transcripts isolated from all *E. granulosus* stages contained the LSM domain present in an RNA-binding protein superfamily involved in pre-mRNA splicing and mRNA processing [61]. Interestingly, a homologue in *Schmidtea mediterranea* (Smed-SmB) is essential for the proliferation of planarian stem cells [62]. Finally, a domain related to bacterial transferase hexapeptide (PF00132), present in a number of transferase protein families [63], appeared expanded in the *E. granulosus* dataset, entirely within the SL library-derived ESTs.

### Secreted proteins appeared only moderately less conserved than non-secreted proteins

Each of the 2,584 peptide predictions (1,848 of which had an initiation methionine) were parsed through the SignalP web server [45], to determine the presence of a putative secretory or anchor sequence. In total 254 peptides (9.8%) were predicted to possess a secretory leader signal (similar to a previous study focusing on *T. solium* larvae [30]), while an additional 157 (6.1%) were predicted to contain a signal anchor. There was no obvious bias to either the GR and SL, or to specific stage libraries (**Table S1**).

**Figure 3:**
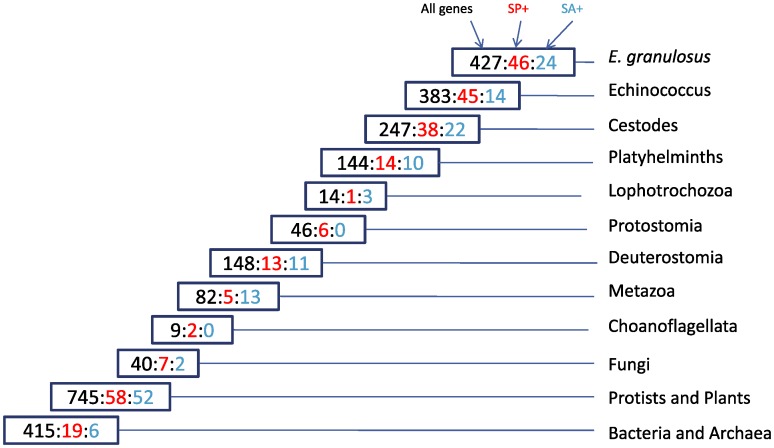
Taxonomic split of *E. granulosus* sequences. For each of the 2,700 *E. granulosus* sequences derived from our clustered dataset, we performed a comprehensive set of BLAST sequence comparisons to a set of 688 partial genomes (see Methods). Using a bit score cutoff of 50, sequences were placed at a node if a sequence match was found in a species dataset associated with that node and not in any more ancient node. The three numbers provided indicate respectively: all sequences; predicted secreted sequences; and predicted membrane anchored sequences. For example, we found that 144 sequences have a BLAST match to a sequence derived from a non-cestode platyhelminth, but not to any species more ancient to the platyhelminths. Of these, 14 are predicted to be secreted and an additional 10 are predicted to be membrane anchored. Note that of the 2,700 putative genes identified in our study, 427 (∼16%) were unique to *E. granulosus*, while an additional 383 (14%) were found to have sequence similarity only to *E. multilocularis*. These findings are consistent with our previous study which shows a high level of genetic diversity even amongst closely related species [147]. Numbers are also consistent with global data indicating that across Eukarya ∼28% of sequences have similarities to protists and plants [147] The tree is based on the phylogenetic analysis by Dunn *et al*. [68].

Previously, in a transcriptomic study of the parasitic nematode *Nippostrongylus brasiliensis*, we noted that signal sequence-bearing proteins showed reduced evolutionary conservation [64]. This observation was confirmed and extended in a subsequent study: parasitic nematodes were found to have a greater proportion of novel, secreted proteins than free-living ones [65]. Here, we examined the conservation of proteins predicted to be secreted within the *E. granulosus* dataset. Based on TBLASTX similarity to partial genomes derived from 688 different eukaryotes, we identified genes/clusters that were unique to *E. granulosus* (15.8%; 14.7% of predicted peptides), specific to *Echinococcus* (30%; 27.7% of predicted peptides), specific to platyhelminths (44.5%) or specific to metazoa (55.2%; **Figure 3**). However, of peptides with a predicted secretory leader sequence, 18.1% were unique to *E. granulosus* and 35.8% were specific to *Echinococcus*. While the former difference is not statistically significant, the latter, being about 30% higher than in the overall dataset, is (p<0.005, Chi-squared test). For signal anchor sequences, the proportions were: 15.3% and 24.2% respectively. While errors in prediction accuracy related to both the SignalP software [45] and truncated sequences may erroneously classify some peptides as containing a secretory sequence, there is no reason to expect that such errors would occur disproportionately amongst the various groups. These results therefore suggest that secreted proteins in *Echinococcus* are less evolutionarily conserved than non-secreted proteins. However, these differences in conservation are much less dramatic than previously reported for *N. brasiliensis*, in which 48.9% of signal positive peptides could be described as genus-specific compared to 26.8% for the dataset overall [64].

### *Echinococcus granulosus* is a platyhelminth

As shown in **Figure 2**, *E. granulosus* is a parasitic cestode and is grouped within the phylum Platyhelminths, along with Trematodes (*e.g.* Schistosoma) and Tricladids (*e.g.* Schmidtea and Dugesia) [66]. Platyhelminths are related to Annelida and Mollusca within the Lophotrochozoa [67], [68]. To investigate the similarity relationships of the genes within our dataset to these various taxonomic groupings, we employed the tool SimiTri [46], that allows simultaneous display and analysis of relative similarity relationships of one dataset to three different databases, to visualize the data from the taxonomic split shown in **Figure 3**.

**Figure 4:**
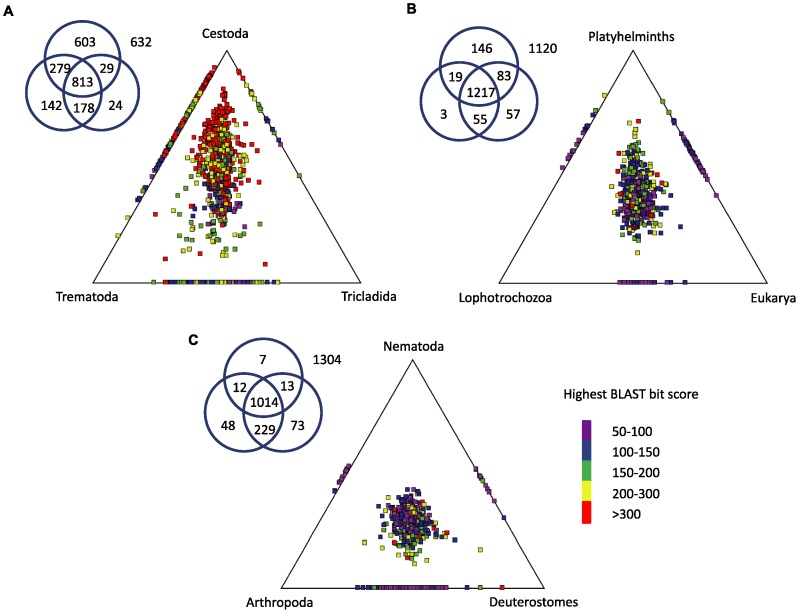
SimiTri relationships of *E. granulosus* sequences. Each plot provides a graphic representation of sequence relationships to three datasets. Each tile in the graphic indicates a unique *E. granulosus* sequence. The closer the tile is to a vertex, the more closely related to a sequence in that dataset relative to the other two datasets. The Venn diagrams show the number of *E. granulosus* sequences associated with each dataset. (A) *E. granulosus* compared with other cestodes, trematodes and tricladids. (B) *E. granulosus* compared with other platyhelminths, other lophotrochozoa (mollusks and annelids) and other eukarya. (C) *E. granulosus* compared with nematodes, arthropods and deuterostomes.

SimiTri analysis showed that *E. granulosus* sequences were, as expected, more closely related to *E. multilocularis* and *T. solium* than to either Tricladids or Trematodes (**Figure 4A**). In addition, very few genes were found to be more similar to a Tricladid species than to a Trematode. This could reflect the closer phylogenetic relationship between Cestodes and Trematodes, which are usually grouped in the Neodermata clade [69]. However, these results may be biased from the larger number of Trematode sequences (74,794) used in this analysis relative to Tricladid sequences (22,327). To examine the impact of sequence coverage, we compared the BLAST score distribution of the *E. granulosus* sequences to randomly selected sets of 22,327 Trematode sequences (Figure S1). This analysis suggests that the higher number of Trematode sequences, rather than the closer relationship between Cestodes and Trematodes, was responsible for the larger number of *E. granulosus* hits to Trematodes compared with Tricladids.

Interestingly, **Figure 4B** shows a relatively low level of enrichment of *E. granulosus* sequences with closer similarity to other Lophotrochozoan (Mollusca and Annelida) sequences than to other Eukaryotes. However, the low level of enrichment for the former may again simply represent a smaller dataset of comparator sequences. Finally, **Figure 4C** shows the relationships to three other major clades of metazoans – Deuterostomia, Nematoda and Arthropoda. The majority of genes showed greater similarity to arthropod and/or deuterostome sequences than to nematode sequences. Given the supporting evidence for the grouping of Nematoda and Arthropoda (Ecdysozoa; [68], [70], [71]), this latter result while potentially indicating the highly diverged nature of nematode genes compared with the other two phyla, nonetheless highlights the limitations of using BLAST sequence similarity scores to infer phylogenetic relationships. See [21], [22], [24], [25], [30] for further discussion on similarity between cestode and trematode datasets and other metazoans.

From the BLAST analyses, we were also able to identify a set of 391 *E. granulosus* genes that shared sequence similarity only with platyhelminths. **Table 3** shows the 34 putative genes that had significant sequence similarity only to four or more other platyhelminth EST datasets. Of these, 19 showed sequence similarity neither to a gene or a protein of known function nor to an identifiable protein domain; of these, five were predicted to be secreted. Only three genes were found to possess a characterized protein domain while 15 showed significant sequence similarity to previously identified or predicted platyhelminth genes with functional annotation. Due to the ubiquity of these gene products within platyhelminths, and although we await their full characterization, they represent a rich source for the identification of potentially novel pan-platyhelminth drug targets.

**Table 3:** Breakdown of pan-platyhelminth *E. granulosus* genes.

	Cestoda		Trematoda					Tricladida							
Cluster	Em	Ts	Cs	Fh	Ov	Sj	Sm	Dj	Dr	ScM	Ml	Domain?	Protein ID	E-value	BLASTX results against NR Description
EGC03065	303	54.7	91.3	60	117	127	127	-	75.1	84.3	68.2	-	CAZ31795.1	2e-33	Dynein light chain (*S. mansoni*)
EGC02854	-	24.3	-	-	-	-	78.6	-	55.5	56.6	68.2	-	CAZ30521.1	5e-18	Disulfide oxidoreductase (*S. mansoni*)
EGC03225	338	300	89.4	53	57.4	58.5	81.3	-	-	62.8	50.4	-	CAZ34857.1	2e-24	Tegumental protein (*S. mansoni*)
EGC03456	370	335	96.3	-	-	84.7	84.3	-	-	-	51.2	-	CAX71449.1	1e-14	Hypothetical protein (*S. japonicum*)
**EGC03443**	307	70.1	-	52	-	60.1	62.4	58.2	65.1	61.2	-	-	AAL14214.1	4e-85	Ag5 precursor (*E. granulosus*)
EGC04874	236	26.2	54.7	-	-	118	51.2	78.6	80.1	74.7	-	-	AAW25970.1	2e-27	SJCHGC09379 protein (*S. mansoni*)
EGC00337	410	207	50.8	-	55.5	50.4	50.8	-	52.4	55.1	-	-	CAX73132.1	7e-05	Calcium-binding EF-hand domain-containing protein (*S. japonicum*)
**EGC03389**	293	274	190	106	-	201	194	66.6	-	97.1	-	-	CAX76877.1	2e-53	Complement C1q-binding protein, mitochondrial precursor (*S. japonicum*)
EGC03454	120	65.9	-	-	90.9	86.7	-	-	60.1	62.8	-	-	CAX69527.1	1e-17	Hypothetical protein (*S. japonicum*)
EGC00482	-	25	-	-	-	100	101	51.6	-	53.1	-	-	CAZ35340.1	2e-18	Hypothetical protein (*S. mansoni*)
EGC01847	-	23.9	-	-	-	154	59.7	97.4	-	52.4	-	-	CAY17707.1	3e-33	Neuroattracting/lsamp/neurotrimin/obcam related cell adhesion molecule (*S. mansoni*)
EGC04177	160	170	67.8	-	61.6	60.5	60.5	-	-	52.8	-	Y	BAG69597.1	1e-38	HSP20 related protein (*E. multilocularis*)
EGC00478	396	337	-	-	-	50.4	50.8	-	55.1	-	-	-	ABK60086	2e-06	Tegumental protein 31.8 kDa (*Clonorchis sinensis*)
**EGC00501**	176	137	-	-	-	81.3	77.4	-	-	65.9	-	-	CAZ34871.1	4e -16	Hypothetical protein (*S. mansoni*)
EGC00718	-	232	-	-	-	106	84.7	-	-	53.1	-	-	ABA40320.1	8e-21	SJCHGC05108 protein (*S. japonicum*)
EGC00791	-	156	-	-	-	127	127	-	-	71.2	-	-	CAZ34108.1	3e-29	Proteasome inhibitor (*S. mansoni*)
**EGC02644**	84.3	130	52.4	74	65.1	63.2	70.9	-	-	-	-	-	CAZ32218.1	9e-14	Hypothetical protein (*S. mansoni*)
EGC02687	129	120	57.4	83	58.2	72.8	70.5	-	-	-	-	-	CAX75643.1	8e-15	Hypothetical protein (*S. japonicum*)
EGC03519	130	119	56.2	78	54.7	67.8	68.9	-	-	-	-	-	CAZ32218.1	7e-16	Hypothetical protein (*S. mansoni*)
EGC02940	214	96.7	62	-	62	52	58.9	-	-	-	-	-	CAZ34970.1	4e-20	Neutral sphingomyelinase (*S. mansoni*)
**EGC03294**	204	107	59.7	-	59.7	52.8	57.8	-	-	-	-	-	CAY17093.1	1e-20	Hypothetical protein (*S. mansoni*)
EGC03628	282	199	-	88	52.4	59.3	54.7	-	-	-	-	-	CAX71798.1	7e-09	Hypothetical protein (*S. japonicum*)
EGC00319	218	207	-	50	58.2	50.1	55.8	-	-	-	-	-	AAX20156.1	5e-53	Tegumental protein (*E. granulosus*)
**EGC00609**	145	55.1	-	62	-	53.9	60.8	-	-	-	-	-	CAZ28306.1	1e-10	Hypothetical protein (*S. mansoni*)
EGC03431	311	23.5	85.5	-	85.1	77.8	92.8	-	-	-	-	-	CAZ34864.1	2e-21	Hypothetical protein (*S. mansoni*)
EGC00292	193	67.8	-	-	-	50.1	58.9	-	-	-	-	-	CAY16950.1	8e-08	Hypothetical protein (*S. mansoni*)
EGC00529	147	23.1	73.6	-	-	69.7	73.2	-	-	-	-	-	AAX28227.2	8e-11	SJCHGC02734 protein (*S. japonicum*)
EGC00534	323	68.2	-	-	-	89.4	89.4	-	-	-	-	-	AAW27475.1	4.e-17	SJCHGC03741 protein (*S. japonicum*)
EGC00981	164	140	-	62	-	70.1	-	-	-	-	-	-	AAW27384.1	1e-11	SJCHGC02564 protein (*S. japonicum*)
EGC01362	196	68.6	-	-	-	65.9	65.5	-	-	-	-	-	CAZ36733.1	1e-09	Hypothetical protein (*S. mansoni*)
**EGC01435**	157	139	-	-	-	67.4	63.2	-	-	-	-	-	CAZ29224.1	3e-11	Isopentenyl-diphosphate delta-isomerase (*S. mansoni*)
**EGC02665**	62	22.3	-	90	-	58.5	59.7	-	-	-	-	Y	CAX70445.1	4e-16	ATPase f0 complex subunit E (*S. japonicum*)
EGC03949	181	25	95.5	-	-	94.4	97.8	-	-	-	-	Y	CAZ30595.1	2e-18	Cytochrome C oxidase copper chaperone (*S. mansoni*)
EGC04938	216	212	-	-	-	62.8	57.4	-	-	-	-	-	-	-	N/A

Pan-Platyhelminth genes were defined as Platyhelminth specific sequences with significant BLAST scores (bit score ≥50) to four or more other platyhelminth EST datasets. The highest BLAST bit scores for each of 11 platyhelminth datasets is shown (consider that a bit score of 50 is roughly equivalent to an e-value of e-5, 100∼e-8, 200∼e-20, etc): Em - *Echinococcus multilocularis*; Ts - *Taenia solium*; Cs - *Clonorchis sinesis*; Fh - *Fasciola hepatica*; Ov - *Opisthorchis viverrini*; Sj - *Schistosoma japonicum*; Sm - *Schistosoma mansoni*; Dj - *Dugesia japonica*; Dr - *Dugesia ryukuensis*; ScM - *Schmidtea mediterranea*; Ml - *Macrostomum lignano*. Clusters with predicted secretory leaders (bold) or signal anchors (underlined) are indicated.

### The properties of SL-bearing transcripts extend currently known aspects of *trans*-splicing in platyhelminths

The SL libraries differed from GR-based libraries in a number of aspects, including a lower level of stage-specificity (**Figure 1B**). Interestingly, a higher overlap of clusters from SL libraries was observed between CW and PSP, the two stages showing comparatively higher metabolic activity, than between PS and either PSP or CW. In addition, as previously noted, a majority of abundant clusters originated from SL libraries (see **Table 2**). As only a fraction of the transcriptome is processed by *trans*-splicing (estimated to be 25–30% in *E. multilocularis* [19], [72]), our equivalent sampling from libraries derived through the two methods (46% GR sequences *vs* 54% SL sequences; see **Table 1**) could explain this bias. However, taking into account that ESTs from either type of library were equally redundant, the previous observations may indicate that a set of *trans*-spliced transcripts is indeed highly expressed in all surveyed stages.

Altogether, 187 clusters, representing 21 ESTs from GR-based libraries and 1,428 ESTs from SL-based libraries, were found to possess a full SL sequence at the 5′end (**Table S1**). Ligation of the GR oligo to the 5′ spliced leader (SL) was observed in the case of highly expressed transcripts (*e.g.* EGC00373 and EGC00435 in **Table 2**). In addition, oligo-capped transcripts lacking SL were found in clusters corresponding to genes that are usually *trans*-spliced (*e.g.* EGC00369 and EGC00647 in **Table 2**). These transcripts could correspond to molecules not yet *trans*-spliced *in vivo*; or to genes that can be expressed with or without the SL [19]. Regarding the latter possibility, it is noteworthy that high-throughput sequencing of the SL *trans*-spliced transcriptome of the tunicate *Ciona intestinalis* revealed that the conventional dichotomy of ‘*trans*-spliced’ *vs* ‘non-*tran*s-spliced’ genes should be supplanted by a view recognizing frequently and infrequently *trans*-spliced genes categories [73].

The set of clusters possessing a full SL sequence allowed us to further characterize *E. granulosus* SL bearing transcripts. Because a conserved and unique feature of flatworm SLs is the presence of a 3′end AUG able to serve as an initiation methionine *in vivo* [74], we analyzed whether the SL ATG was in frame with the major ORF of the cDNAs and, furthermore, what proportion of these was full-length. Of the 187 SL-bearing clusters, 143 were predicted to be full length, using the ATG in the SL as the putative start codon (8 of these are listed in **Table 2** together with 6 where the SL ATG is not in frame with the predicted ORF). It is likely that not all *E. granulosus trans*-spliced transcripts actually use the SL AUG *in vivo*, as alternative AUGs were often found within a few codons of the SL AUG. This was the case, *e.g.* in 4/8 cDNAs listed in **Table 2** (an additional ATG was present within 5 codons 3′of the SL); however, in the remaining 4 cDNAs, the SL AUG would be required as an initiation methionine if the N terminus was to fully correspond to those of phylogenetically conserved orthologous proteins. Thus, our data provide additional evidence that the SL AUG could serve as an initiation methionine in platyhelminths, as indicated by earlier studies in this phylum [19], [74], [75], [76]. Moreover, we searched for *E. granulosus* orthologs of 35 *S. japonicum* genes known to be both expressed by *trans*-splicing and using the SL AUG as an initiation methionine [74]. Putative orthologs (BLAST bit score ≥100; or >40% identity over at least 90% coverage) were identified for 16, 15 of which were derived from SL libraries; of these, 10 would use the SL AUG as an initiation methionine, indicating that the use of *trans*-splicing and initiation from the SL AUG is itself phylogenetically conserved in the Neodermata.

We then examined the potential functional relationships between the products encoded by different *trans*-spliced mRNAs. No particular functions or processes were found to be enriched within *trans*-spliced cDNAs, in agreement with previous reports in other flatworms [19], [75], [76], [77], including a recent study that identified a large set of *trans*-spliced genes in *S. mansoni* using high-throughput sequencing (11% out of ∼11,000; [78]). In contrast, and as was described for tunicates [73], [79], [80], genes encoding ribosomal proteins tended not to be *trans*-spliced (see **Table S1**).

### A set of long non-protein coding RNAs was dominant in all three stages

Although polypeptides could be predicted from 95.7% of the clusters, the remaining 116 clusters appeared to be non-protein coding. Quite strikingly, a majority (66) of these – accounting for ∼700 ESTs mostly from GR libraries of the three stages – contained segments displaying high identity (≥90%) with fragments of EgBRep, a previously described middle repetitive DNA element from *E. granulosus*, showing structural similarities to mobile elements [55]. Some of these clusters were relatively abundant (notably, EGC00310 and EGC03058; see **Table 2**; and also EGC02905, EGC02701, EGC00351, EGC00367 and EGC01002, all with ≥20 ESTs; see **Table S1**). Collectively, the ESTs within these clusters represented >10% of sequences from each stage.

**Figure 5:**
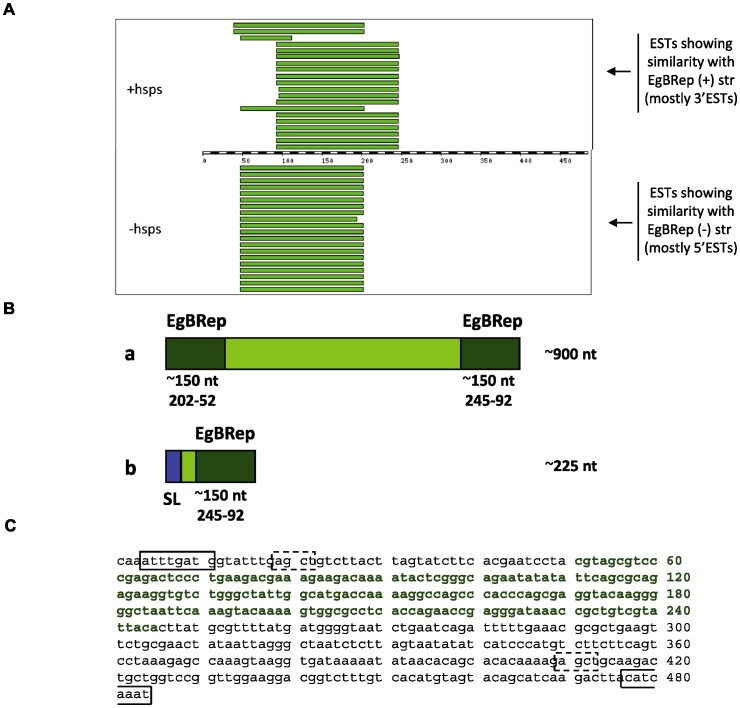
Analysis of dominant non-protein coding transcripts. (A) Fragments of the EgBRep repetitive element are present at the 5′ and 3′ends of non-protein coding transcripts. Graphical output of the BLAST analysis of EgBRep (484 bp) *vs. E. granulosus* ESTs at the *Echinococcus* blast server (available at: http://www.sanger.ac.uk/cgi-bin/blast/submitblast/Echinococcus). The diagram shows the EST regions with significant BLAST scores to sense (+hsps) and antisense (−hsps) EgBRep. Most sequences showing similarity to the sense strand (over the fragment ∼90–245 nt) were 3′ESTs, whereas those showing similarity to the antisense strand (∼50–200 nt) were 5′ESTs. (B) Molecular architecture of EgBRep containing transcripts. Schematic representation of: (a) the dominant transcripts identified in all GR libraries; (b) the putatively *trans*-spliced transcript sequenced mainly from the PSSL library. The fragments with similarity to EgBRep are indicated in dark green; the central tracts showing microdiversity in individual sequences in light green; and the sequence of the SL spliced-leader in blue. The sequence of EgBRep [55] is shown in (C) to illustrate the overlap of the fragments homologous to the 5′ (from 92 to245 nt) and 3′ends (from 52 to 202 nt) of the dominant transcripts that led to the artificial concatamerization of ESTs in the original assembly. The fragment between positions 52 and 245 is marked in dark green, terminal inverted repeats are boxed with solid lines, and *Alu*I restriction sites with dashed lines. See the text and **Table S2** for further details.

The assembled sequences of clusters EGC00310 and EGC03058 corresponded to full-length transcripts of ∼900 nt, putatively capped and polyadenylated (as shown by the presence of the GR oligo at the 5′end and poly(dA) at the 3′end in non-trimmed sequences). These transcripts matched the minus strand of EgBRep over ∼150 nt at both the 5′ and 3′ends (**Figures 5A and 5B**). Moreover, multiple reads mapping between these conserved flanking sequences showed microdiversity in the central tract, reaching a global identity of about 90%. Manual assembly of the EgBRep-containing ESTs, avoiding artificial collapse of contigs by the automated algorithm (see **Figure 5C**), identified two clusters, named Cluster A (512 ESTs, including all but 4 of the ESTs from the original clusters EGC00310 and EGC03058) and Cluster B (187 ESTs) (see **Figure 5B** and **Table S2**). Interestingly, some EgBRep-containing sequences were *trans*-spliced (notably, those in EGC02791; see **Tables 2** and **S1**). These were almost exclusively from the PS library and corresponded to *trans*-spliced polyadenylated transcripts of ∼225 nt that included the 150 nt 3′end fragment similar to EgBRep (see **Figure 5B** and ClusB.contig10 in **Table S2**).

Comparison of these consensus sequences to the current version of the *E. granulosus* genome (available at http://www.sanger.ac.uk/cgi-bin/blast/submitblast/Echinococcus) identified scaffolds showing regions of high identity (90–100%) with the manually assembled contigs, and revealed that some of them are likely to derive from transcripts processed by *cis*-splicing (*e.g.* ClusB.contig8 has 2 exons, and ClusB.contig7 has 3 exons). For every EgBRep-containing contig, several highly similar fragments (>80% identity) were present in the draft genome.

Transcripts with similarity to EgBRep were also identified in *E. multilocularis* ESTs from an oligo-capped metacestode library, including presumed orthologs of the abundant *E. granulosus* transcripts derived from EGC00310 and EGC03058, with an overall similarity between *Echinococcus* spp. of 92% (see *e.g.* clusters EMC00034 and EMC00190 in PartiGeneDB). Moreover, abundant, putatively non-protein coding cDNAs, showing scattered segments of 85–100% identity with the *E. granulosus* EgBRep-containing cDNAs, were present in the *T. solium* transcriptome (∼6,100 clusters available at PartiGeneDB; see *e.g.* TSE00132, TSE00439 and TSE00790).

The occurrence of these EgBRep-containing cDNAs in all surveyed stages is a major feature of the larval transcriptome of *E. granulosus*. Structurally, these transcripts correspond to a class of long (>200 nt) non-protein coding RNAs (ncRNAs), first described during the large scale sequencing of mouse full-length cDNA libraries [81], that resemble mRNAs (being capped, polyadenylated and often spliced), yet lacking clear open reading frames. Recent genome-wide studies have identified large numbers of long ncRNAs in human and model organisms [82], [83], [84], [85], [86], [87] and shown that some of them overlap with repeats [82], [83], [85], [87], and that short conserved regions nested in rapidly evolving sequences are present in long ncRNAs conserved between species (see *e.g.* [82], [85], [87]). In addition, some *C. elegans* primary long ncRNAs have been found to be *trans*-spliced [87]. Long ncRNAs have been implicated in the regulation of gene expression through a variety of mechanisms (reviewed by [88], [89]) and were found to participate in stem cell pluripotency and differentiation [90]. In addition, an appreciable portion can be processed to yield small RNAs ([84]; reviewed by [89]).

Because EgBRep-containing transcripts are associated with repeats, they could be precursors of piRNAs, a class of strikingly diverse small RNAs implicated in transposon silencing in the metazoan germ-line (reviewed by [91]). piRNAs are likely generated via processing of long single-stranded precursors (primary piRNAs), transcribed by RNA polymerase II from discrete genomic loci (piRNA clusters), some of which are highly enriched in transposons and other repeats (reviewed by 91,92). Notably, a long ncRNA associated with an insect transposable element has been proposed to be the precursor of rasiRNAs [93], a class of piRNAs first identified in *Drosophila melanogaster* [94]

In recent years, the piRNA pathway has emerged as a distinctive trait of planarian somatic stem cells (neoblasts) and piRNAs were found to predominate among small RNAs in the neoblasts of *S. mediterranea* [95], [96]. Neoblasts are the only mitotically active cells in planarians; they are responsible for their extraordinary regenerative capacity and are known to also give rise to germ-line stem cells (reviewed by [97]). In the Neodermata, and in cestodes in particular, there is evidence that similar mechanisms of self-renewal exist ([98], [99]; reviewed by [54]). It remains to be determined, therefore, whether EgBRep-containing long ncRNAs are themselves active molecular species or represent precursors of small RNAs; in the latter case, they could be precursors of piRNAs in proliferating cells from each of the parasite materials sampled in our study.

### Fermentative pathways appear to be up-regulated in the germinal layer

**Figure 6:**
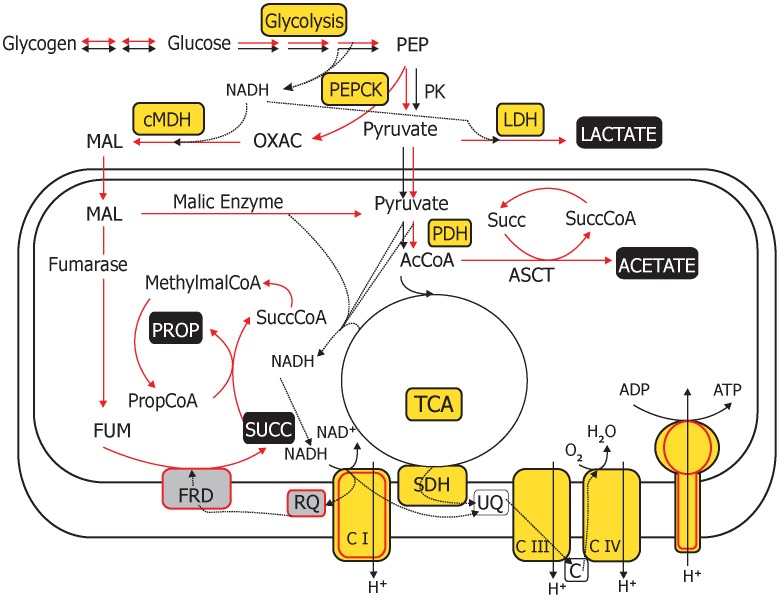
Main pathways of carbohydrate catabolism in parasitic flatworms with special reference to *E. granulosus* (adapted from [**148**] **).** Aerobic pathways are indicated by black arrows and anaerobic pathways by red lines; enzymes or pathways found in the *E. granulosus* transcriptome are in yellow and additional components identified in *E. multilocularis*, in grey [149]; end products of fermentation routes are in black with white letters (acetate and propionate are also marked because they have been observed as excreted end products of *E. granulosus* metabolism [102]; although enzymes for their generation were not found in our dataset). Mitochondrial fermentation via malate dismutation branches out from glycolysis at the level of PEP, which is converted into oxalacetate and the latter into malate. In the mitochondria, malate is dismutated to pyruvate and succinate, a conversion first catalyzed by the TCA enzyme fumarase, and then by the membrane-associated fumarate reductase. This is an electron transport-complex, which oxidizes rhodoquinol to rhodoquinone; the latter is recycled to rhodoquinol by complex I. Since fumarate, which is the final electron acceptor, is generated endogenously, the whole pathway is fermentative, although it is sometimes considered as anaerobic respiration. It produces 4–5 mol ATP/mol glucose (depending on whether succinate is further catabolyzed to propionate), more energy than that obtained from glycolysis (2 mol ATP/mol glucose). If aerobic glycolysis was also involved in energy production, some pyruvate would enter the TCA cycle, whereas a majority would be converted to lactate, thus generating ∼4 mol ATP/mol glucose [105]. Abbreviations: AcCoA, acetyl-CoA; ASCT acetate:succinate CoA-transferase; C, cytochrome c; CI-IV, complexes I to IV of the respiratory chain; FRD, fumarate reductase; FUM, fumarate; LDH, lactate dehydrogenase; MAL, malate; cMDH, cytosolic malate dehydrogenase; Methylmal-CoA, methyl malonyl-CoA; OXAC, oxalacetate; PDH, pyruvate dehydrogenase; PEPCK, phosphoenol pyruvate carboxy-kinase; PK, pyruvate kinase; PROP, propionate; Prop-CoA, propionyl-CoA; RQ, rhodoquinone; SDH, succinate dehydrogenase; SUCC, succinate; Succ-CoA, succinyl-CoA; TCA, tricarboxylic acid cycle; UQ, ubiquinone.

Genes in several key energy production pathways were differentially expressed in the surveyed stages, with fermentation predominating in CW, and gluconeogenesis being up-regulated in CW and PSP (**Table 4**). The data are consistent with the previously reported existence of a complete tricarboxylic acid (TCA) cycle in *E. granulosus* [100], [101]. Genes encoding components of respiratory complexes I, III and IV were also identified, indicating that aerobic respiration can take place in the surveyed stages (**Table 4**, **Figure 6**).

**Table 4:** Enzymes involved in energy metabolism.

Enzyme	Cluster ID	ESTs	% SL	CW	PS	PSP
Glycolysis and pyruvate decarboxylation						
Fructose-bisphosphate aldolase	EGC00369	44	9	26	8	10
Glyceraldehyde 3-phosphate dehydrogenase	EGC00305	8	0	4	2	2
Phosphoglycerate mutase	EGC03341	4	0	–	–	4
Enolase beta subunit	EGC04828	1	0	–	1	–
Enolase alpha subunit	EGC03002	1	0	–	1	–
Pyruvate dehydrogenase E1 component subunit alpha type II	EGC05022	1	0	1	–	–
Pyruvate dehydrogenase E1 component subunit beta type II	EGC04914	1	0	1	–	–
Pyruvate dehydrogenase, dihydrolipoamide acetyltransferase component	EGC00336	1	0	1	–	–
TCA cycle and mitochondrial complexes*						
Citrate synthase	EGC00287	3	0	3	–	–
Isocitrate dehydrogenase [NAD] subunit gamma	EGC01292	15	100	13	1	1
2-oxoglutarate dehydrogenase E1 component	EGC00395	4	100	2	–	2
Succinate dehydrogenase iron-sulfur protein (Complex II)	EGC00994	1	0	–	1	–
NADH-ubiquinone oxidoreductase chain 1 (Complex I)	EGC00089	2	100	–	2	–
NADH-ubiquinone oxidoreductase chain 4 (Complex I)	EGC00090	3	33	–	3	–
NADH dehydrogenase [ubiquinone] 1 alpha subcomplex subunit 8 (complex I)	EGC02944	4	0	–	4	–
NADH dehydrogenase 1 alpha subcomplex subunit 5 (Complex I)	EGC00596	3	100	–	–	3
NADH dehydrogenase [ubiquinone] Fe-S protein 8 (Complex I)	EGC04834	1	0	–	1	–
NADH-ubiquinone oxidoreductase B18 subunit (Complex I)	EGC03592	1	0	–	–	1
NADH-ubiquinone oxidoreductase ashi subunit (Complex I)	EGC00965	1	0	–	1	–
NADH-ubiquinone oxidoreductase Fe-S protein 2 (Complex I)	EGC01705	1	100	1	–	–
Ubiquinol-cytochrome c reductase, Rieske Fe-S protein (Complex III)	EGC00324	6	0	6	–	–
Cytochrome b-c1 complex subunit 8 (Complex III)	EGC01165	4	0	–	2	2
NADH-cytochrome b5 reductase (Complex III)	EGC03367	3	0	–	–	3
Cytochrome c oxidase subunit 1 (Complex III)	EGC03652	1	0	–	–	1
Cytochrome c oxidase subunit 2 (Complex IV)	EGC00086	2	0	–	–	2
Cytochrome c oxidase subunit IV (Complex IV)	EGC00897	2	0	1	–	1
Cytochrome c-type heme lyase	EGC00912	1	0	1	–	–
ATP synthase subunit beta, mitochondrial	EGC04244	1	0	1	–	–
Fermentation# (homolactic and malate dismutation)						
Lactate dehydrogenase, chain A	EGC00284	22	0	22	–	–
	EGC04966§	1	0	1	–	–
	EGC00302§	1	0	1		
Malate dehydrogenase (cytosolic)	EGC00028	3	0	3	–	–
Phosphoenolpyruvate carboxykinase[Table-fn nt111a] 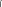 (3 fragments, C- to N-terminus)	EGC04068	6	0	6	–	–
	EGC04111	2	0	2	–	–
	EGC03250	5	0	4	1	–
Gluconeogenesis						
Fructose-1,6-bisphosphatase, isoform B	EGC00659	24	100	12	1	11
	EGC01761§	1	100	1	–	–
Glycogenolysis and glycogenesis						
Phosphoglucomutase	EGC01351	4	100	4	–	–

* Some enzymes of the TCA cycle (e.g. fumarase) and mitochondrial complex I can also be considered as part of the fermentation pathways (see the text and legend to Figure 6); for simplicity, they are included in the former category only.

# Cluster EGC00753 (CW: 16; PSP: 5) encodes a mitochondrial citrate lyase beta-like protein, which could be involved in citrate fermentation.

§ Clusters corresponding to incompletely processed transcripts (*i.e.* they contain non-removed introns).

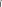
Also participates in gluconeogenesis.

Some enzymes belonging to key fermentation pathways coupled to glycolysis were also found (**Figure 6**). In particular, cytosolic fermentation to lactate appeared to be an important metabolic route in the germinal layer: lactate dehydrogenase (LDH) was highly expressed in the CW. In addition, transcripts for phosphoenol pyruvate carboxykinase (PEPCK) and cytosolic malate dehydrogenase (cMDH) were also present (mainly in CW libraries), indicating the existence of a route for mitochondrial fermentation via malate dismutation (**Figure 6**), which is an unusual feature of helminth metabolism. The existence of these fermentative pathways is consistent with the fact that lactate and succinate were described as the major end-products of carbohydrate metabolism [102].

In addition, enzymes for gluconeogenesis (fructose-1,6-bisphosphatase; and also PEPCK), glycogenolysis and glycogenesis were also found (**Table 4**), in agreement with the accepted view that glucose is the major respiratory substrate and glycogen the main energy store molecule in flatworms [102].

Considered globally, the germinal layer appears to possess a high metabolic activity (see **Table 4**), involving, in particular, fermentative pathways. The synthesis of the laminated layer towards the outside of the cyst and the generation of brood capsules containing PS towards the inside are major metabolic demands for the germinal layer, of both energy and intermediate metabolites. It is possible that the oxygen supply within the hydatid cyst may be limited by the thick laminated layer. In this respect, it is worth noting that *in vitro* growth of *E. multilocularis* metacestode has been reported to be more active under microaerobic conditions, suggesting metabolic adaptations to low oxygen [103], which may include glycolysis through generation of lactate, and use of the PEPCK-succinate pathway. Alternatively, the up-regulation of lactate fermentation (and malate dismutation) could be due to ‘the Warburg effect’ observed in cancer and all proliferating cells [104], [105]. Indeed, proliferative tissues convert most glucose to lactate through ‘aerobic glycolysis’, regardless of whether oxygen is present; lactate fermentation and other anaerobic pathways are thought to facilitate the uptake and incorporation of nutrients into the biomass (reviewed by [104], [105]; see also **Figure 6**). Interestingly, glutamine synthetase, which is also highly expressed in proliferating tissues, was observed to be an abundant transcript in the CW (and PS; see EGC00519 in **Table S1**). In addition to the essential role of glutamine in protein and nucleotide synthesis, this amino acid is an anabolic substrate. Glutamine can be converted into pyruvate via TCA and glutaminolysis providing biosynthetic carbons for the production of macromolecules [106], [107].

### Thiol and selenol antioxidant enzymes are highly expressed in all larval stages

Parasites must cope with oxidants and reactive oxygen species (ROS) derived from their own aerobic metabolism and also from host activated cells such as phagocytes. Several redox-based antioxidant enzymes were present in all surveyed stages, and many of them were highly expressed (**Table 5**). Peroxiredoxins (Prx, formerly known as thioredoxin peroxidases), glutathione peroxidase (GPx), thioredoxin (Trx), selenoprotein W, glutaredoxin (Grx) and methionine sulfoxide reductase (Msr) were among the 7% most highly expressed genes. A cytosolic Prx was particularly abundant in the CW, while expression of Gpx, Msr-b (stereospecific for the Met-S-sulfoxide), selenoprotein W and the Trx-related EGC00370 increased upon pepsin/H^+^ PS activation. Although Cu/Zn superoxide dismutase(s) are known to be expressed in both PS and CW [108] we did not identify corresponding clusters in our data. We also failed to identify any clusters corresponding to catalase transcripts, confirming previous reports of absence of catalase activity in *E. granulosus* and other flatworms (reviewed by [109]). Globally, the data indicate that a broad range of antioxidant defences are dependent on the enzyme thioredoxin glutathione reductase (TGR), which functions as a metabolic hub for transferring electrons to glutathione (GSH), Trx, Grx and from these latter to their targets, such as Prx, Msr, GPx, etc (reviewed by [109], [110]). Although TGR was absent from the dataset (which may be due to the fact that it is encoded by a long mRNA, of 2.8 kb), all known direct and indirect targets of this enzyme were present.

**Table 5:** Antioxidant and detoxification enzymes.

Enzyme	Function	Cluster ID	No ESTs	% SL	CW	PS	PSP
Mn superoxide dismutase (mitochondrial)	Superoxide dismutation to hydrogen peroxide and oxygen	EGC00326	2	50	1	–	–
Peroxiredoxin (cytosolic)	Trx-dependent hydrogen peroxide reduction	EGC00084 EGC02722*	36	0	20	8	8
		EGC05011#	1		1		
		EGC01022#	1			1	
Peroxiredoxin (mitochondrial)	Trx-dependent hydrogen peroxide reduction	EGC00918	10	90	4	–	6
Glutathione peroxidase	GSH-dependent hydrogen peroxide reduction	EGC00127	10	10	1	3	6
Thioredoxin (cytosolic)	Protein disulfide reduction	EGC00470	11	0	–	7	4
Thioredoxin related (monodomain Trx, lacks the canonical CGPC active site)	Protein disulfide reduction	EGC00370	52	100	18	12	22
Thioredoxin (mitochondrial)	Protein disulfide reduction	EGC01178	4	100	2	–	2
Glutaredoxin (mitochondrial) (monodomain Grx, monothiolic)	Protein-GSH disulfide reduction, Fe/S assembly and transfer	EGC00387	13	100	5	5	3
Glutaredoxin (monodomain Grx belonging to the Grx PICOT-like family, monothiolic)	Protein-GSH disulfide reduction, Fe/S assembly and transfer	EGC03379	1	0	–	–	1
Methionine sulfoxide reductase R (Msr-a)	Trx-dependent methionine sulfoxide reduction (R-stereospecific)	EGC01853	4	25	3	–	1
Methionine sulfoxide reductase S (Msr-b)	Trx-dependent methionine sulfoxide reduction (S-stereospecific)	EGC00562	15	100	1	4	10
Selenoprotein W	GSH-dependent antioxidant, precise function unknown	EGC00635	14	0	1	5	8
Glutathione S-transferase (mu class)	GSH transfer to electrophiles (see also text)	EGC00080	2	0	–	–	2
Glutathione S-transferase (microsomal)		EGC01588	3	33	1	1	1
		EGC03483#	1	0			1
Glutathione S-transferase (sigma-like class)	Detoxification, reduction of lipid peroxides, synthesis of prostaglandins and leukotrienes	EGC04109	4	0	4	–	–
Glutathione S-transferase (sigma-like class)	Detoxification, reduction of lipid peroxides, synthesis of prostaglandins and leukotrienes	EGC03317	1	0	–	1	–

* EGC00084 and EGC02722 encode the same protein (there is a difference in one nucleotide between them, most likely due to an artifact); the number of ESTs provided is the total from both clusters.

# Clusters corresponding to incompletely processed transcripts (*i.e.* they contain non-removed introns).

Many eukaryotic selenoproteins are important antioxidant enzymes with higher turnover rate than their Cys homologs. *E. granulosus* TGR is known to be a selenoenzyme [111] and the GPx and selenoprotein W transcripts we detected also contain an in-frame UGA codon and a SECIS (Selenocysteine insertion sequence) element [112]. However, the Msr-b is a Cys-containing protein and not a selenoprotein, as is the case of one of the isoforms present in mammals [112].

In addition to acting as direct and indirect antioxidant, GSH also serves a detoxification role through glutathione *S*-transferases (GSTs). These enzymes are primarily involved in detoxification of electrophiles, but many of them possess additional or distinct functions, including the neutralization of oxidative stress (through *e.g.* removal of lipid peroxides, inactivation of secondarily oxidized products and regeneration of *S*-thiolated proteins), as well as the catalysis of metabolic reactions not involved in detoxification (*e.g.* biosynthesis of leukotrienes and prostaglandins) (reviewed by [113], [114]). Four distinct GSTs belonging to different families and classes were present in our dataset. Three belong to the family of cytosolic GSTs: two are of sigma class and one corresponds to the previously characterized mu-class enzyme [115]. The last one belongs to the microsomal GST family. Although the precise functions of these GSTs remain to be determined, sigma-class GSTs have been mostly implicated in prostaglandin synthesis [113], [114].

### A set of apomucin-encoding genes is highly expressed in the germinal layer

**Figure 7:**
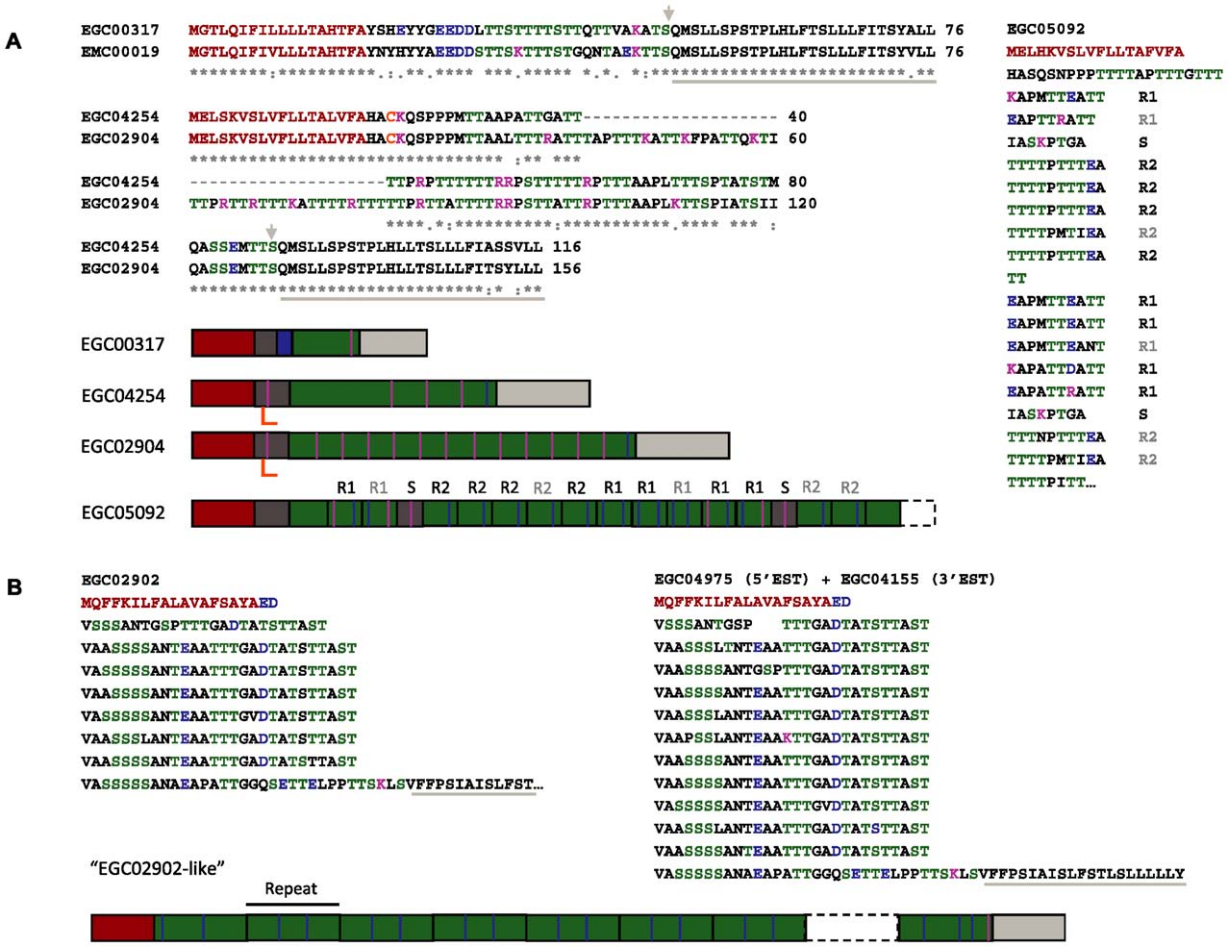
Apomucin-encoding clusters in the cyst wall transcriptome. Molecular organization of apomucins expressed by the CW and not found in PS and PSP (A); or with homologs in the other stages (B). The alignments in (A) show the full-length sequences of the proteins predicted from EGC00317 and its putative *E. multilocularis* ortholog (EMC00019); and of EGC2904 and its shorter variant EGCO4254. Fully conserved residues are marked with (*), those replaced with amino acids of strongly similar properties with (:) and of weakly similar properties with (.). The sequences predicted from: EGC05092 in (A); EGC02902 and the manually assembled overlap of EGC4155 and EGC4975 (forward and reverse sequences of the same cDNA clone) in (B) are also included, in a format that highlights the tandem repeats identified in their mucin cores. The residues forming the predicted signal peptides are in brown and the C-terminal extensions putatively corresponding to a signal for the addition of a GPI anchor (A) or forming a transmembrane helix (B) are marked with light grey lines (the ω sites predicted by the PI predictor are indicated in (A) with an arrow of the same color). Some amino acids of the N-terminal extensions and mucin cores of the mature apomucins are shown in colors: Ser/Thr predicted to be *O*-glycosylated in green, Asp/Glu in blue, Arg/Lys in magenta and unpaired Cys in orange. A schematic of the same features is included below the sequences using identical colors. See the text and **Table 6** for further details. R1, R2 and S represent: Repeat 1, Repeat 2 and Spacer in EGC05092; imperfect repeats are indicated in grey.

Several clusters coding for apomucins were identified in the larval transcriptome on the basis of a high Ser/Thr content offering multiple potential *O*-glycosylation sites consistent with mucin synthesis. A set of 4 apomucins expressed by the CW were not found in PS and PSP, whereas a second set (16 clusters) were present in all assayed materials (**Figure 7** and **Table 6**).

**Table 6:** Apomucin-encoding clusters in the CW transcriptome.

Cluster ID	No. ESTs	Mature apomucin			*O*-Glyc	Specific features
		N-term	Core	C-term		
Clusters with no homologs in other stages*						
EGC00317	37 GR	Acidic	T-rich	Signal for GPI addition	11 T+3 S	C-term extension almost identical to EGC02904 and EGC04254
EGC02904	13 GR	Unpaired Cys	T-A-R/K-P-rich	Signal for GPI addition	50 T+5 S	C-term extension almost identical to EGC00317
EGC04254 (variant of EGC02904)	6 GR	Unpaired Cys	T-A-R/K-P-rich	Signal for GPI addition	30 T+6 S	C-term extension almost identical to EGC00317
EGC05092	3 GR	Related to EGC02904 (no Cys)	Two types of 10 aa repeats	Lacks C-term	∼every T	Repeat1: XAPM/ATTXATT (X = acidic/basic). Repeat2: TTTTPTTTEA. Spacer: IASKPTGA.
Clusters with homologs in other stages#						
EGC02902	5 GR	Short, acidic	7 repeats	Lacks C-term§	∼15 S/T per repeat	Repeats of 28 aa: T/S-A-rich with interspersed D and E
EGC04971	5 GR	Lacks N-term	≥11 repeats	Lacks C-term		
EGC04155[Table-fn nt117] 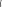	1 GR	Lacks N-term	5 repeats	Signal for TM helix		
EGC04975[Table-fn nt117] 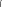	1 GR	Short, acidic	7 repeats	Lacks C-term		

* An additional cluster contains ESTs from CW only (EGC01419, 2 CWSL); it is not included because the available sequence lacks its N- and C-terminus.

# Clusters with ESTs from PSGR: EGC03003; EGC03208; EGC03329; EGC04734; EGC04753; EGC04824. Clusters with ESTs from PSPGR: EGC02726; EGC02761; EGC02775 (includes 1 EST from CWGR); EGC03388; EGC03397; EGC03487.

§ Most likely, 7 C-terminal amino acids (see Figure 7B).

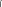
ESTs from these clusters are forward and reverse sequences of the same clone.

The CW apomucins have a distinct structure. Three (EGC00317, EGC02904 and EGC04254) were the most highly expressed protein-coding transcripts of the germinal layer altogether (4% of ESTs from the CWGR library, with EGC00317 accounting for 2.6%; **Table 2**). These feature no tandem repeats, contain a very high proportion of putative *O*-glycosylation sites with interspersed basic residues and a common C-terminal sequence that is predicted to correspond to a signal for the addition of glycosylphosphatidylinositol (GPI) anchors. Two of them (EGC02904 and EGC04254) may be splice or allelic variants of each other (they differ mainly by a 40 amino acid insertion in the mucin core), and carry unpaired Cys residues in their N-terminal extension. The fourth CW apomucin (EGC05092) has the same N-terminus as the proteins predicted from EGC02904 and EGC04254 but it has a distinct mucin core with two different tandemly repeated units of 10 amino acids. All four apomucins have a marked predominance of Thr over Ser residues, suggestive of secreted mucins.

Interestingly, a putative ortholog of EGC00317 was identified among *E. multilocularis* ESTs from an oligo-capped metacestode library (see EMC00019 at PartiGeneDB and **Figure 7A**). The overall identity between the predicted *Echinococcus* spp. apomucins was 84%; it was high (>95%) over the signal peptide and C-terminal sequence, but surprisingly low for putative orthologs of these organisms over the rest of the molecule (∼63%).

This family of apomucins could form the backbones of the mucins from the fibrilar component of the laminated layer, a unique *Echinococcus* structure whose synthesis is known to be a major metabolic activity of the germinal layer, as was recently proposed in a comprehensive review of this structure [13]. The high level of expression of these apomucins and the existence of an ortholog in the transcriptome of *E. multilocularis* metacestodes support this inference. In addition, Thr is known to be the most abundant amino acid of laminated layer preparations (reviewed by [13]), consistent with the preponderance of this residue in the predicted mature apomucins (**Table 6**). Finally, in agreement with intense mucin biosynthesis, a number of CW clusters encode enzymes and transporters involved in the assembly of *O*-glycans (**Table 7**). In particular, probably reflecting the marked predominance of galactose in the major glycans purified from the laminated layer [116], several transcripts correspond to proteins participating in galactose metabolism, the synthesis of UDP-galactose and its translocation across Golgi membranes.

**Table 7:** Proteins involved in the synthesis of *O*-glycans in the CW transcriptome.

Cluster ID	No. ESTs	Predicted function (from blast similarity)
EGC00399*	2 SL	UDP-GalNac:polypeptide GalNAc transferase (first step in the synthesis of *O*-glycans)
EGC04989	3 GR	
EGC01546	2 SL	Core 1 β1–3 galactosyltransferase (elongation of core 1 with Gal β1–3)
EGC04121	1 GR	
EGC00364#	7 SL	β1–4 galactosyltransferase
EGC00902	2 SL	UDP-glucose 4-epimerase (galactose metabolism)
EGC01356	2 SL	Gal-1-phosphate uridylyl transferase (synthesis of UDP-galactose)
EGC00933	2GR/1 SL	UDP-galactose transporter

* Already characterized: Eg-ppGalNAc-T1 [151].

# Also contains ESTs from PSSL (7) and PSPSL (6) libraries.

The second set of mucin-encoding transcripts (EGC02902 and related clusters in **Figure 7B** and **Table 6**) include a very short acidic N-terminus followed by a varying number of tandemly repeated units of 28 amino acids. These repeats each contain two acidic residues and about 15 Ser/Thr (Ser/Thr ratio ∼0.8), all of which would be glycosylated. The C-terminal extension ends with a stretch predicted to be a transmembrane helix, indicating that they are cell-surface proteins. These mucins could thus be constituents of the mucin coat known to cover the tegument of larval and adult worms [17]. The presence of transcripts from these genes in the CW libraries could derive from apomucin expression in the germinal layer or from developing PS in the tissue of the CW.

### Several members of the tetraspanin family are expressed in the surveyed stages

Fourteen clusters encoded members of the tetraspanin family (TSP, **Figure 2**) and some of them were among the most abundant in the dataset (notably, EGC00290 and EGC00446; **Table 2**). TSPs are a large family of highly expressed type II membrane proteins (200–350 amino acids) with a characteristic topology (four transmembrane domains; small and large outer loops, short N- and C-terminal tails). They have conserved disulfide bridges in the large extracellular loop (LEL) that are the basis of a structural classification ([117]; reviewed by [118]).

Eleven *E. granulosus* TSPs (EgTSPs; **Table 8**) showed substantial similarity to TSPs from *E. multilocularis* (Em-TSPs; [119]) and *T. solium* (TsT-24; [120]); (see **Figure S2**). Two EgTSPs (EGC00709 and EG04933) were most similar to schistosome TSPs. EgTSP EGC04745 was not classified with other flatworm TSPs and was most similar to an insect TSP. Some transcripts likely encode variants of the same TSP (proteins predicted from the two contigs in EGC00097 share 94% identity, and EGC00817 and EGC03391 share 93% identity), as has been observed in schistosomes [121].

**Table 8:** Members of the tetraspanin family in the larval transcriptome.

Cluster ID – Blast similarity to UniProt/EMBL	No. ESTs	CW	PS	PSP	Length (aa)	Cys in LEL*
EGC00446 - B6VFH3 – *E. multilocularis* TSP-1–263 aa [e-140, 95% identity - 251/263 aa]	34 GR	–	21	13	263	6
EGC00290 - B6VFH3 – *E. multilocularis* TSP-1–263 aa [7e-81, 49% identity - 128/260 aa]	29 GR	29	–	–	263	6
EGC00129 - B6BFH7 – *E. multilocularis* TSP-5–225 aa [e-122, 97% identity - 218/225 aa]	28 GR	16	6	6	225	6 CD63-L
EGC03207 (incompletely processed form of transcript in EGC00129)	1 GR	–	1	–	–	–
EGC00097 - B6VFH3 – *E. multilocularis* TSP-1–263 aa - Ctg 1 [2e-26, 29% id - 75/261 aa]	4 GR	2	2	–	250	6
EGC00097 - B6VFH3 – *E. multilocularis* TSP-1–263 aa - Ctg 2 [3e-27, 30% identity - 76/261 aa]	7 GR	–	–	7	250	6
EGC00299 - B6VFH3 – *E. multilocularis* TSP-1–263 aa [2e-09, 28% identity - 70/254 aa]	9 GR	9	–	–	262	6
EGC00643 - B6VFH8 – *E. multilocularis* TSP-6–222 aa [e-121, 98% identity - 217/222 aa]	6 GR	–	4	1	221	4
EGC02782 (incompletely processed form of transcript in EGC00643)	1 GR	–	–	1	–	–
EGC00817 - Q5GM22 – *T. solium* T-24–225 aa [1e-90, 71% identity - 161/226 aa]	5 GR	5	–	–	226	6 CD63-L
EGC03391 - Q5GM22 – *T. solium* T-24–225 aa [3e-89, 70% identity - 159/226 aa], from aa 79, 96% identical to E. multilocularis TSP-3# [142/148 aa]	5 GR	2	1	1	226	6 CD63-L
EGC04251 - B6VFH5 – *E. multilocularis* TSP-3–148 aa# [2e-41, 80% identity - 65/81 aa]	2 GR	2	–	–	81 (C-term)	6
EGC04959 - B6VFH5 – *E. multilocularis* TSP-3–148 aa# [2e-32, 84% identity - 65/77 aa] Predicted protein identical to C-term of EGC00817	1 GR	1	–	–	77 (C-term)	6
EGC00709 - Q5DB78 – *S. japonicum* § – 291 aa [5e-83, 62% identity - 144/230 aa]	1 GR	1	–	–	231 (lacks N-and C-term)	8
EGC04933 - P27591 – *S. japonicum* Sj-23–218 aa [2e-29, 38% identity - 66/173 aa]	1 GR	1	–	–	208 (lacks C-term)	4
EGC00849 - B6VFH3 – *E. multilocularis* TSP-1–263 aa [1e-12, 25% identity - 47/190 aa]	1 SL	1 (no SL)	–	–	183 (lacks N-term)	6
EGC04745 - EFN81996 – *Harpegnathos saltator* CD151 antigen – 241 aa [6e-10, 32% identity – 43/135 aa]	1 GR	–	1	–	157 (lacks N-term)	6
**Total no. ESTs**		69	36	29		

**Figure 8:**
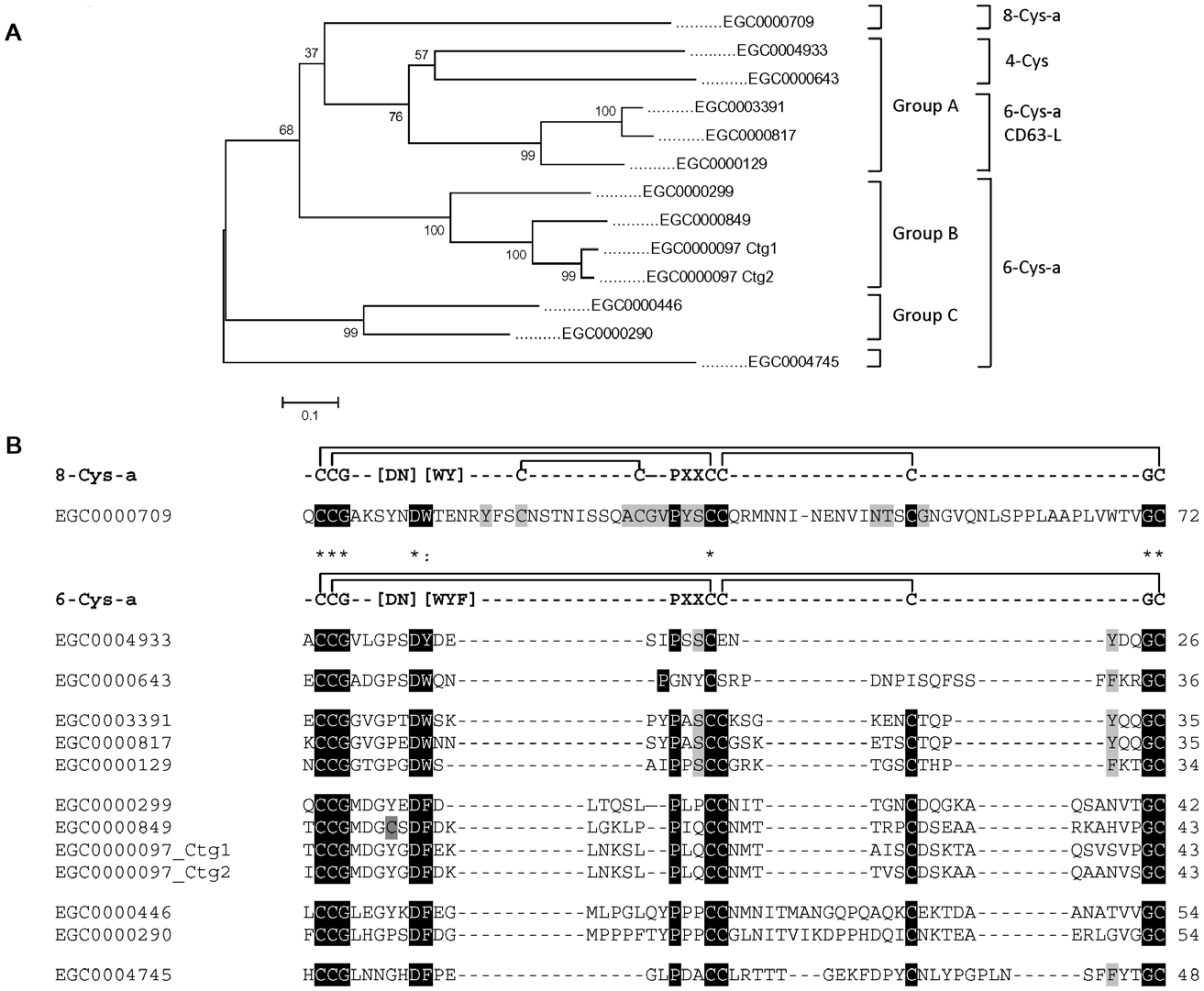
Members of the tetraspanin family in the larval transcriptome. (A) Phylogenetic analysis of *E. granulosus* TSPs. The phylogenetic tree was constructed with twelve EgTSPs; the identified groups and the LEL Cys pattern (see below) are indicated on the right. The sequences translated from EGC04251 and EGC04959 were excluded because only a C-terminal fragment is available for both of them. See **Table 8** for further details. (B) Cys pattern of the LEL variable domain of EgTSPs. The figure shows an alignment of the hypervariable regions of the twelve EgTSPs analyzed in (A), manually refined taking into account the consensus of 6-Cys-a and 8-Cys-a cysteine patterns (adapted from [49] and [50]). Fully conserved residues are marked with (*) and a conservative replacement with (:). Consensus residues present in individual sequences are marked in white on black shading; conserved amino acids in CD63-like and TSPAN15-like TSPs present in EgTSPs conforming, respectively, to the 6-Cys-a and 8-Cys-a patterns are shaded in light grey. The canonical topology of disulfide bonds is shown above each consensus. Note that: i) EGC00643 and EGC04933 lack Cys4 and 5; ii) EGC00643 is unusual in having ‘PXXXC’ instead of ‘PXXCX’; it was aligned considering that Cys3 is fully conserved; iii) EGC00849 is unusual in having an extra Cys in the LEL variable domain (shaded in dark grey).

* Number of Cys residues in LEL (large extracellular loop; see Figure 8B).

# The sequence reported for EmTSP-3 [119] is only 148 aa-long and lacks the canonical TM domains 1 and 2 of the TSP family. The residue assigned as initiation methionine corresponds to Met present between TM2 and TM3 in EmTSP-5 (see Figure S2).

§ Sj-TSP-26, according to Wu *et al.* [121].

Phylogenetic analysis of the EgTSPs identified three clades (**Figure 8A**). Group A includes two close paralogs (the variants EGC00817 and EGC03391, and EGC00129, with 67% identity), and two more distant proteins. Group B comprises three proteins, including another pair of close paralogs (the variants from EGC00097 and EGC00849, with >70% identity). A third pair of close paralogs forms a separate group (Group C; EGC00290 and EGC00446, with 48% identity), while the remaining two EgTSPs (EGC00709 and EGC04745) appear quite distant from the rest, especially the one with no flatworm homolog.

Alignment of the LEL variable region of EgTSPs highlighted their Cys patterns and, in some cases, allowed assigning them to specific groups (**Figure 8B**). Most EgTSPs have 6 Cys in their LEL and conform to the 6-a pattern [49], [50]. In addition, some Group A EgTSPs show structural features present in CD63-like TSPs [49]; interestingly, these EgTSPs also contain a putative tyrosine-based sorting signal (YXXΦ, where Φ is a bulky, hydrophobic residue), which is known to be involved in CD63 intracellular trafficking (reviewed by [122]; see **Figure S2**). The other EgTSPs from Group A have only 4 Cys. It is likely that, as described for other animal TSPs, Cys 4 and 5 were secondarily lost in these proteins [49], [50]. Group B and Group C EgTSPs and the one predicted from EGC04745 also have a 6-Cys-a pattern but they lack other structural features of CD63-like TSPs and their LELs are longer. Finally, EGC00709 encodes a TSP with 8-Cys-a pattern and conforming to the TSPAN15-like group [49]. CD63- and TSPAN15-like EgTSPs have been identified in all metazoan groups ([49], [123]; see also [121]).

A majority of EgTSPs were expressed in the CW, some of them at high levels (in particular, EGC00290 from Group C, EGC00299 from Group B, EGC00817 and EGC00129 from Group A). EGC00446 (Group C) and EGC00643 (Group B) included ESTs derived solely from PS and PSP libraries (**Figure 8A** and **Table 8**). A similar level of developmentally regulated transcription was recently reported for schistosome TSPs [121].

Most of the EgTSPs identified in our dataset represent cestode expansions of the family. Indeed, excepting two proteins, they are considerably distant even from trematode TSPs. This observation supports the hypothesis that gene duplication and rapid divergence have been major driving forces in the evolution of TSPs, where lineages are phylum-specific and many genes appear to be species-specific [50], [121], [124]. Interestingly, distinct members from the identified groups would be up-regulated in particular stages. TSPs regulate migration, fusion and signaling by acting as organizers of multimolecular membrane complexes involving the plasma membrane, intracellular vesicular compartments and exosomes (reviewed by [118] and [125]). Novel TSPs may thus have evolved to fulfill the highly diverse requirements of distinct parasite stages. In this context, it is worth noting that TSPs have been assayed as vaccine antigens for schistosomiasis [126], [127] and primary alveolar echinococcosis [119] in mouse models. In both systems, some level of protection was observed upon immunization with particular TSPs. Mammalian TSPs involved in highly specific functions are also amenable to targeting using antibodies, with considerable therapeutic potential against various pathologies (reviewed by [128]).

### Different AgB subunits predominated in the germinal layer and protoscoleces

Three clusters sharing sequence similarity with *E. granulosus* antigen B (AgB) were identified within our dataset: EGC00327, EGC00450 and EGC03328. AgB is a highly abundant lipoprotein present in hydatid fluid [129]. It is the most relevant antigen for hydatid disease diagnosis (see *e.g.* [130]) and has been associated with a number of immunomodulatory functions in the host [131]. AgB has been extensively characterized at the protein [5], [132], [133] and gene levels (see *e.g.* [4], [134]); and its physiological lipid ligands have recently been described [135]. EGC00450 and EGC03328 with 21 and 14 ESTs respectively, derived exclusively from PSGR and PSPGR libraries. They corresponded to virtually identical AgB3 variants that differ only in the length of the acidic stretch. The third cluster, EGC00327 with 8 CWGR ESTs, corresponded to AgB4. These findings indicate a clear bias in the expression of AgB3 and AgB4 subunits in the different parasite materials.

Remarkably, no ESTs encoding AgB1 or AgB2 were found in our dataset. These subunits were originally cloned from PS [136], [137], and the corresponding cDNAs have subsequently been detected by several authors, mainly in PS (see *e.g.* [138], [139], [140]).

Two studies, on *E. granulosus* [134] and *E. multilocularis* [141], have reported developmentally regulated expression of AgB subunits in the *Echinococcus* life cycle, using real-time PCR and semi-quantitative PCR, respectively. Both included material from the germinal layer and the adult stage; but resting PS were only assayed in *E. multilocularis* [141] and pepsin/H^+^-activated PS only in *E. granulosus* [134]. The two studies found that AgB1, B2, B3, and B4 were expressed in the CW. AgB4 was expressed at lower levels than the other subunits, and was most highly expressed in CW. AgB1 and B3 predominated in PS [141], whereas AgB3 was highly dominant in PSP [134] and adult worms [134], [141] (the latter also expressed some AgB5 [134], [141]). If we assume that expression in PS is similar between *Echinococcus* spp., our data on AgB3 and AgB4 are consistent with these reports. In contrast, the absence of cDNAs corresponding to AgB1, B2 and B3 in the CW library, and to AgB1 in the PS library appear to contradict the previous observations. We hypothesized that the discrepancy could derive from the oligo-capping procedure, which is known to exclude transcripts whose 5′UTRs do not efficiently ligate to the oligo-cap [18]. To explore this possibility, we cloned cDNAs from AgB1–AgB4 obtained by RACE or RLM-RACE: no difference was detected in cloning efficiencies for the transcripts of the different genes. The analysis of the 5′UTR from oligo-capped cDNAs showed the presence of different numbers of GT repeats in AgB1–AgB4 subunits, which did not appear to interfere in the cloning procedure. AgB1 was the most expressed gene in the germinal layer and AgB3 in PS, while AgB2 was the least expressed in both stages (A. Arend and A. Zaha, unpublished). Consequently, we have no explanation as to why AgB1 encoding ESTs were absent from our dataset.

### Concluding remarks

Although cestodes are a major group of parasites of humans and animals, extensive genomic coverage has only recently begun for these organisms [4]. Key advances have been made with transcriptomics for several platyhelminths, including mainly parasitic trematodes (see *e.g.* [20], [21], [22]) and the planarians *S. mediterranea* [142], [143], [144]; and *Dugesia japonica* [145], to which we can now add our gene discovery project on the dog tapeworm *E. granulosus*. This has fulfilled our objectives of greatly expanding the information available on genes expressed by larval parasites, and of identifying a series of candidate molecules involved in the host-parasite cross-talk in hydatid infections.

The new data we present in this report provide insights on many important biological features of this fascinating parasitic organism. Firstly, *E. granulosus* follows an elaborate developmental program through its life cycle that relies on the activity of somatic stem cells (reviewed by [54]). The highly expressed long ncRNAs we have identified may be involved in the regulation of gene expression through that program in response to environmental cues in the host. In addition, we have identified a number of genes reflecting specificities of particular stages including those whose expression is up-regulated by pepsin-acid activation. Regarding these latter, a major finding was the identification of a family of Kunitz-type serine protease inhibitors associated mostly with pepsin/H^+^-treated PS, which we have previously described [146]. Another major finding relates to the metabolic activity needed to maintain the intermediate host interface. Indeed, we found clear signs of enhanced energy production in the germinal layer and identified several genes that could form the mucin backbones of the laminated layer, as well as enzymes involved in their glycosylation.

Secondly, we have identified numerous new potential genes for investigation, either because they are highly expressed by the parasitic larvae and are novel in sequence, or because by sequence similarity to genes of known function they are attractive candidates for drug targeting. The generation of effective new pharmaceuticals is critically important for both *Echinococcus* species (and also for *T. solium*), which cannot be controlled by current agents and which therefore can develop life-threatening infections [1].

Thirdly, the dataset richly illustrates the dynamics of multigene family evolution in platyhelminths, both with respect to selective expansion of particular families and with regards to the subset bearing predicted signal peptides. At this stage, before the completion of the genome, gene family expansion at the transcriptomic level could represent either or both gene multiplication and diversification, or elevated expression of a similar repertoire of gene variants. In either instance, certain gene families are clearly of emphasized importance in *E. granulosus*.

Finally, because ESTs were derived from full-length enriched cDNA libraries prepared from carefully selected parasite materials, our data will constitute a high quality complement of the full genome sequence of the parasite, now nearing completion [4]. Indeed, preliminary sequence comparisons found that 94% of our predicted consensus sequences could be mapped to the current draft genome of *E. granulosus* (>90% identity over >80% consensus sequence length – data not shown).

### Accession numbers

The *E. granulosus* ESTs generated in this work were deposited in dbEST with the following accession numbers: BI243991-BI244549; BQ172910-BQ173849; BU582013; CN648894-CN653840; CV223690-CV223699; CV678041-CV681224; CV678546; CV678796.

## Supporting Information

Figure S1 **BLAST bit score distribution of Trematode and Tricladid matches to** ***E. granulosus*** **sequences.** Graphs indicate the number of *E. granulosus* matches to three different datasets: i) all Trematode sequences (74,794 sequences); ii) all Tricladid sequences (22,327 sequences); and iii) 22,327 randomly selected Trematode sequences (100 samples – standard deviation shown). Note the large increase in matches with a BLAST bit score <50 when the number of Trematode sequences is reduced to a similar level as the Tricladid sequences. These results indicate that the larger number of sequences associated with the Trematode dataset was responsible for the apparent closer relationship between Cestodes and Trematodes visualized in **Figure 4A**.
(TIF)

Figure S2 **Comparison of** ***E. granulosus*** **and related cestode tetraspanins.** Full-length EgTSPs identified in our dataset were aligned with highly similar proteins from *E. multilocularis* (Em-TSP1, 5 and 6) and *T. solium* (Ts-T24, the ortholog of Em-TSP5; [120]). Fully conserved residues are marked with (*), those replaced with amino acids of strongly similar properties with (:) and of weakly similar properties with (.). The residues of the LEL variable region that are conserved in 6-Cys-a TSPs are marked in white on black shading, and those present in the sub-family of CD63-like TSPs are shaded in light grey [49], [50]. The residues forming a putative tyrosine-based sorting signal at the C-terminus of CD63-like TSPs are marked in white on dark grey shading [122]. The position of the transmembrane domains (TM1–TM4, boxed) was determined by TMHMM analysis and manually adjusted according to the study of Kovalenko *et al* [150]. Where necessary, the sequences of EgTSPs were edited taking into account the results of BLAST analysis and the original EST traces. Accession numbers of the cestode TSPs in Uniprot/EMBL are as follows: Em-TSP1, 5 and 6, B6VFH3, 7 and 8, respectively; TsT-24, Q5GM22.
(EPS)

Table S1 **Summary of** ***E. granulosus*** **EST clusters.**
(XLS)

Table S2 **Manually assembled contigs from EgBRep containing ESTs.**
(XLS)
