# Correction: A Transcriptomic Analysis of *Echinococcus granulosus* Larval Stages: Implications for Parasite Biology and Host Adaptation

**DOI:** 10.1371/annotation/984cd209-4e98-467c-b462-c11090e43be7

**Published:** 2012-12-04

**Authors:** John Parkinson, James D. Wasmuth, Gustavo Salinas, Cristiano V. Bizarro, Chris Sanford, Matthew Berriman, Henrique B. Ferreira, Arnaldo Zaha, Mark L. Blaxter, Rick M. Maizels, Cecilia Fernández

In the author list, the "¤a" superscript following the name of John Parkinson should instead follow James D. Wasmuth. Furthermore, the address linked with this symbol is as follows:

Department of Ecosystem and Public Health, Faculty of Veterinary Medicine, University of Calgary, Calgary, Canada

