# Supplementary figures and images for: A Transcriptomic Analysis of Echinococcus granulosus Larval Stages: Implications for Parasite Biology and Host Adaptation
Source: PLoS Negl Trop Dis. 2012 Nov 29;6(11):e1897. doi: 10.1371/journal.pntd.0001897 (PMC3510090; doi:10.1371/journal.pntd.0001897)

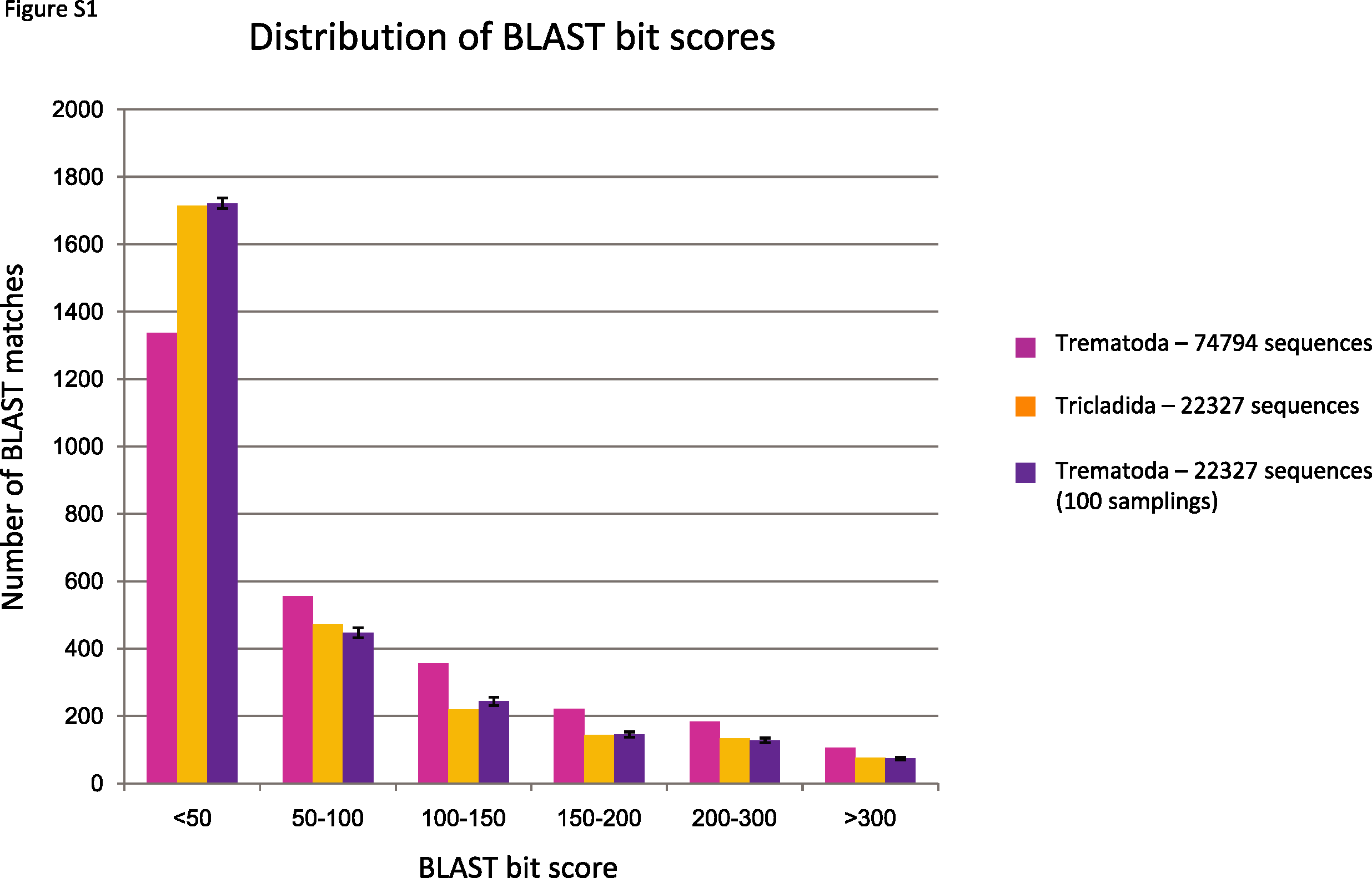

Supplement: Figure S1 — BLAST bit score distribution of Trematode and Tricladid matches to E. granulosus sequences. Graphs indicate the number of E. granulosus matches to three different datasets: i) all Trematode sequences (74,794 sequences); ii) all Tricladid sequences (22,327 sequences); and iii) 22,327 randomly selected Trematode sequences (100 samples – standard deviation shown). Note the large increase in matches with a BLAST bit score <50 when the number of Trematode sequences is reduced to a similar level as the Tricladid sequences. These results indicate that the larger number of sequences associated with the Trematode dataset was responsible for the apparent closer relationship between Cestodes and Trematodes visualized in Figure 4A . (TIF) [file pntd.0001897.s001.tif]
